# Evolutionary Origin of the Mitochondrial Cholesterol Transport Machinery Reveals a Universal Mechanism of Steroid Hormone Biosynthesis in Animals

**DOI:** 10.1371/journal.pone.0076701

**Published:** 2013-10-04

**Authors:** Jinjiang Fan, Vassilios Papadopoulos

**Affiliations:** 1 Research Institute of the McGill University Health Centre, Montréal, Québec, Canada; 2 Department of Medicine, Biochemistry and Pharmacology and Therapeutics, McGill University, Montréal, Québec, Canada; University of Nevada School of Medicine, United States of America

## Abstract

Steroidogenesis begins with the transport of cholesterol from intracellular stores into mitochondria *via* a series of protein-protein interactions involving cytosolic and mitochondrial proteins located at both the outer and inner mitochondrial membranes. In adrenal glands and gonads, this process is accelerated by hormones, leading to the production of high levels of steroids that control tissue development and function. A hormone-induced multiprotein complex, the transduceosome, was recently identified, and is composed of cytosolic and outer mitochondrial membrane proteins that control the rate of cholesterol entry into the outer mitochondrial membrane. More recent studies unveiled the steroidogenic metabolon, a bioactive, multimeric protein complex that spans the outer-inner mitochondrial membranes and is responsible for hormone-induced import, segregation, targeting, and metabolism of cholesterol by cytochrome P450 family 11 subfamily A polypeptide 1 (CYP11A1) in the inner mitochondrial membrane. The availability of genome information allowed us to systematically explore the evolutionary origin of the proteins involved in the mitochondrial cholesterol transport machinery (transduceosome, steroidogenic metabolon, and signaling proteins), trace the original archetype, and predict their biological functions by molecular phylogenetic and functional divergence analyses, protein homology modeling and molecular docking. Although most members of these complexes have a history of gene duplication and functional divergence during evolution, phylogenomic analysis revealed that all vertebrates have the same functional complex members, suggesting a common mechanism in the first step of steroidogenesis. An archetype of the complex was found in invertebrates. The data presented herein suggest that the cholesterol transport machinery is responsible for steroidogenesis among all vertebrates and is evolutionarily conserved throughout the entire animal kingdom.

## Introduction

The rate-limiting step in steroid biosynthesis is the transport of the sole substrate cholesterol from intracellular stores into mitochondria where cholesterol is metabolized by the inner mitochondrial membrane enzyme cytochrome P450 family 11 subfamily A polypeptide 1 (CYP11A1) to pregnenolone, which is the precursor of adrenal, gonadal, placental, and brain steroids [1]. In adrenal glands and gonads, cholesterol transfer into mitochondria is accelerated by hormones and cAMP, leading to the production of high levels of steroids that reach all tissues and cells of the body through circulation. These steroids control multiple body functions during the lifespan of the organism. In placenta and brain, steroid synthesis is required for control of local tissue functions, although no hormonal regulation has been identified.

**Figure 1:**
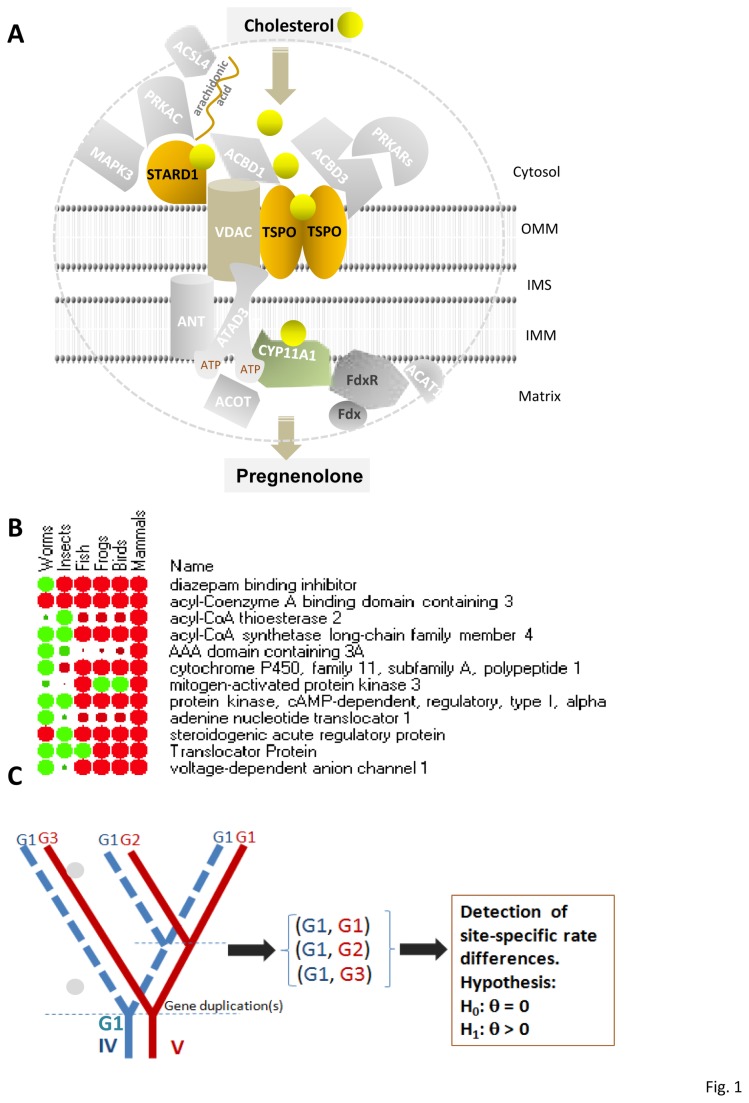
Machinery for mitochondrial cholesterol transport for steroidogenesis and functional prediction. A. Schematic diagram of the transduceosome, steroidogenic metabolon, and associated signal transduction proteins and their likely roles in mitochondrial cholesterol transport and cholesterol metabolism into the first intermediate product, pregnenolone, during steroidogenesis. Each member and the closest members in their corresponding gene family were selected for further evolutionary analysis. IMM, inner mitochondrial membrane; IMS, intermembrane space; OMM, outer mitochondrial membrane. B. Orthologous origin of human TSPO-mediated steroidogenesis complex proteins in other animals. The proteins include Translocator Protein 18-kDa (TSPO) previously known as peripheral benzodiazepine receptor (PBR or BZRP), steroidogenic acute regulatory protein (STAR) or START domain containing 1 (STAR/STARD1), acyl-CoA binding domain containing 3 or PBR- and PKA-associated protein 7 (ACBD3/PAP7), cytochrome P450, family 11, subfamily A, polypeptide 1 (CYP11A1), acyl-CoA binding domain containing 1 or diazepam binding inhibitor (GABA receptor modulator, acyl-CoA binding protein; ACBD1/DBI), protein kinase, cAMP-dependent, regulatory, type I, alpha (PRKAR1A), ATPase family, AAA domain containing 3A (ATAD3), mitogen-activated protein kinase 3 or extracellular signal-regulated kinase ½ (MAPK3/ERK1/2), acyl-CoA synthetase long-chain family member 4 (ACSL4), acyl-CoA thioesterase 2/3 (ACOT2/3), voltage-dependent anion channel 1 (VDAC1), adenine nucleotide translocator 1 or solute carrier family 25 (mitochondrial carrier; adenine nucleotide translocator), member 4 (ANT1/SLC25A4). BLAST expectation value (*e*-value), a statistical calculation based on the score that gives the number of hits that this search would return by chance in a genome database, was used in the ranking of the best matches (“hits”) from the database. A higher e-value may suggest a distant evolutionary relationship. The max identity (percent similarity between the query and subject sequences over the length of the coverage area) was used to show the occurrence of the proteins in the organism as ink blots of the heatmap where the diameter of each dot corresponds to the value. That is, the ink blot is smallest exactly halfway between the minimum and maximum values and becomes progressively larger as the values approach the minimum or maximum. Green to red indicates the value (%) from smaller to bigger. The data was normalized in each row. C. The *in* *silico* prediction of functional divergence in protein evolution by site-specific rate shifts using the maximum likelihood method in DIVERGE [29]. G1-G3, paralogous gene number 1 to 3; IV, invertebrate; V, vertebrate; H_0_ and H_1_, hypothesis of the type I evolutionary functional divergence using a coefficient of statistical divergence (θ), which is the change in function that resulted from different rates at these sites between clusters. If the null hypothesis of θ = 0 was not rejected, the evolutionary rate is virtually the same between two clusters of genes, and this result implies that these clusters share the same function(s). The dotted line indicates that gene duplication did not occur.

Many years of research have focused on the search for a specialized cholesterol transport protein which will bring free cholesterol from intracellular stores, mainly lipid droplets but also plasma membrane, to mitochondria, and allow for its segregated incorporation in the outer mitochondrial membrane, movement thought the aqueous intramitochondrial membrane space and loading onto CYP11A1 at the matrix side of the inner mitochondrial membrane. A series of proteins essential for steroid hormone formation (Figure 1A), including the mitochondrial translocator protein (18kDa; TSPO), cytosolic steroidogenic acute regulatory protein (STAR) or START domain-containing 1 (STARD1), acyl-coenzyme A binding domain-containing 3 (ACBD3, PAP7), a protein interacting directly with TSPO, acyl-coenzyme A binding domain-containing 1 (ACBD1) or diazepam-binding inhibitor (DBI), an endogenous TSPO ligand, and cAMP-dependent protein kinase regulatory type I alpha (PRKAR1A), which is critical for the phosphorylation for STARD1 to induce STARD1 activity, in part, at the outer mitochondrial membrane [2–5].

Over the past few years, however, it became clear that none of these proteins acted alone to achieve this process and that cholesterol transport occurs through a series of sequential protein-protein interactions involving these cytosolic proteins as well as mitochondrial proteins located at both the outer and inner mitochondrial membranes [2,6,7]. Thus, we previously identified a hormone-induced multiprotein complex composed of the above mentioned cytosolic and outer mitochondrial membrane proteins, TSPO, and voltage-dependent anion channel (VDAC1) that control the rate of cholesterol entry into the outer mitochondrial membrane, and we termed this complex the transduceosome [8]. More recently, a bioactive, multimeric protein complex that spans the outer-inner mitochondrial membranes was found to be responsible for the hormone-induced import, segregation, targeting, and metabolism of cholesterol by the inner mitochondrial membrane CYP11A1. This steroidogenic metabolon allows for the fast and efficient transport and targeting of the substrate cholesterol to its site of metabolism without equilibration, interference and diffusion with the surrounding mitochondrial environment. This metabolon includes TSPO, VDAC1, the AAA domain-containing 3A (ATAD3A), CYP11A1, and its cofactors ferredoxin (FDX) and ferredoxin reductase (FDXR)/adrenodoxin reductase (AdR; Figure 1A) [5–7,9]. Obviously, TSPO and VDAC1 are integral parts of both the transduceosome and metabolon and form the connection between these two complexes.

In addition to these proteins, several signaling proteins were shown to be critical for this first step of steroid biosynthesis. These proteins include the mitogen-activated protein kinase 3 (MAPK3, also known as extracellular signal-regulated kinases [ERK1/2]), which is required for phosphorylation of STARD1, acyl-CoA synthetase long-chain family member 4 (ACSL4), and acyl-CoA thioesterase 2/3 (ACOT2/3) (Figure 1A) [10,11]. As noted earlier, one of the main mitochondrial components, VDAC1, was identified as part of both the transduceosome and the steroidogenic metabolon, whereas the role of the inner mitochondrial membrane adenine nucleotide translocase type 1 (ANT1) remains questionable in this process [7,12–14]. Thus, the cholesterol transport machinery includes the transduceosome and the steroidogenic metabolon as well as the relevant signal transduction molecules.

Most of the work on steroidogenesis has been carried out in either mice or humans, and both discrepancies and similarities have been reported in other animal models, such as zebrafish [15]. Considering that cholesterol is an important component of cell membranes and lipoproteins in all animals, its use as the sole precursor of steroid formation seems to have some unique features in nearly all animals compared to other organisms, such as plants, where cholesterol is usually a minor constituent of the sterol fraction, and most single cellular microbios [16,17]. This notion raises the possibility that the established mechanism of steroidogenesis has general implications for the entire animal kingdom. Cholesterol has undergone an adaptive evolution to allow multicellular organisms to cope with the hazards of oxygen [18]. The evolution of the molecular chemistry from lanosterol to cholesterol has been shown to be driven by an increase in the ability of the sterols to promote and stabilize the liquid-ordered phase, which is related to domain formation and to its structure and function in biological membranes [19]. Sterol biosynthesis pathways have been identified in all eukaryotic phyla and even in some bacteria [20]. Additionally, a comprehensive view of the evolution of major enzymes of the steroid hormone biosynthesis pathway in animals reveals that cholesterol metabolism to various steroid products has evolved independently in three lineages: arthropods, nematodes, and vertebrates. Furthermore, the steroidogenic cytochrome P450 enzymes evolved from enzymes that detoxify xenobiotics [21]; however, an ancestor of the progesterone chemoreception signal transduction system in invertebrates was shown to be comparable to the advanced assimilation of sexual behavior in mammals [22]. Whether an ancestral cholesterol transport complex exists in animals other than humans and mice remains unknown.

In a recent study, we reported that the *Tspo* gene family, which was present in the last universal common ancestor dating 5 billion years ago, was expanded during evolution from an environmental sensor or signal transducer to a functional bioregulator adapted to organism-, tissue-, cell-, and organelle-specific needs [23]. We proposed that the function of TSPO in oxygen-mediated metabolism was the primary role of this protein during evolutionary history, facilitating evolution of specific roles in cholesterol trafficking and steroid hormone biosynthesis in animals and other organisms [23].

This comprehensive analysis on the TSPO protein family in combination with the availability of massive genome sequence data from a variety of eukaryotic organisms inspired us to apply phylogenomic approaches to study the origin and evolution of the mitochondrial cholesterol transport machinery. Here, we report that the evolutionary origin of members of the steroidogenesis machinery involved in cholesterol transport can be traced back to invertebrates, including worms and insects, and therefore, an “archetype” of the complex is present in all animal phyla. In addition, this machinery exists in all vertebrates, suggesting a common rate-limiting first step in steroidogenesis, despite the occurrence of some gene duplication events during early vertebrate evolution.

## Materials and Methods

### Sequence retrieval

Most studies on cholesterol transport of steroidogenesis machinery members were performed in mice and humans (Figure 1A). Thus, not all annotated genes are available for other organisms. Using BLASTP, we searched the National Center for Biotechnology Information (NCBI) public non-redundant databases to retrieve homologous genes for the steroidogenesis machinery members involved in cholesterol transport. We used the relevant query for each protein from mouse (http://blast.ncbi.nlm.nih.gov/Blast.cgi; Table S1). The top 500 protein sequences were used for further screening of selected genes and primary amino acid sequence alignments.

### Multiple sequence alignments

We aligned the top 500 translated sequences from each dataset using ClustalW [24] and manually edited the alignments to remove the partial and/or not well-aligned sequences in BioEdit [25]. The remaining sequence sets were aligned again using ClustalW with manual sequence alignment edition by eye. These alignments were imported in FASTA format for further sequence processing.

### Phylogenetic analysis

The evolutionary history of each gene family was estimated by phylogenetic analysis using a neighbor-joining (NJ) method as implemented in the molecular evolutionary genetics analysis software version 4.0 (MEGA4) with options of pairwise deletion and Dayhoff PAM (point accepted mutation) matrix model [26,27]. All positions containing alignment gaps and missing data were eliminated in the pairwise sequence comparisons (pairwise deletion option). The evolutionary distances were computed using the Dayhoff matrix-based method [28]. Bootstrap support values for NJ trees were obtained from 1000 replicates, and only bootstrap values larger than 50% are shown next to the branches. The tree is drawn to scale, and the horizontal length of each branch is given in the same units as those of the evolutionary distances used to infer the phylogenetic tree. The distances are provided in units of the number of substitutions per amino acid site.

### Functional divergence analysis

To estimate the functional divergence after the gene duplication events, we used a maximum-likelihood method implemented in the Diverge program [29,30]. This approach was used to test whether a significant change in the rate of evolution (type I functional divergence) had occurred by estimating the log-likelihood value of the hypothesis by assuming a value for the coefficient of functional divergence (θ > 0) and comparing this likelihood with that under the hypothesis of no functional divergence (θ = 0). The likelihood-ratio test was performed, and this test can be approximated to a χ^2^ distribution with 1 degree of freedom. A θ that is significantly greater than 0 indicates altered selective constraints of amino acid sites during the protein family evolution (Figure 1B).

We used this method to trace the functional origin of the cholesterol transport members in the steroidogenesis machinery from vertebrates back to invertebrate animals. Selected sequences from each duplicated member and the members before gene duplication events were used for multiple protein sequence alignment using ClustalW [24]. A customized phylogenetic NJ tree from MEGA4 [27] was used as an input to select each protein category of interest.

### Protein homology modeling and molecular docking

We predicted the putative three-dimensional structure of STAR proteins via an automated comparative protein modeling server (Swiss-Model) (http://www.expasy.ch/spdbv) at the University of Geneva with the optimized mode using the coordinates of human MLN64 protein (1EM2) available from the Brookhaven Protein Database (BPD) [31,32]. A total of 13 sequences, including STAR and STARD3, from humans to worms/insects were aligned using ClustalW. The evolutionary conservation scores were calculated using a Bayesian method implemented in the ConSurf web server (http://consurftest.tau.ac.il). The scores were mapped onto the three-dimensional structure of a STAR homology model using the ConSurf web server [33]. The three-dimensional structure of human CYP11A1 was used to illustrate its relationship to the inner mitochondrial membrane as well as to the putative cholesterol recognition/interaction amino acid consensus (CRAC) domain [34] and steroid binding pocket [35]. A three-dimensional protein threading model of *C. elegans* CYP44A1 was generated using human CYP11A1 as a template in Swiss-PDB viewer (version 4.1) [36]. Molecular docking analysis was performed using AutoDock-vina [37], and the ligand PDB coordinates were obtained from ChemSpider (http://www.chemspider.com) and converted by OpenBabel.

## Results

### Identification of the homologous cholesterol transport members involved in the steroidogenesis machinery using the basic local alignment search tool (BLAST)

The orthologous origin of the twelve members of the human TSPO-mediated steroidogenesis complex proteins were identified in other animals in search of the NCBI GenBank databases using the basic local alignment search tool (BLAST) and mouse orthologous genes as queries (Figure 1A). The BLAST expectation values (*e*-values), which are calculated based on the number of hits that this search would return by chance, were used to show the likelihood that these proteins are present in that organism. The lower the *e*-value, the more significant the score; however, a higher *e*-value may suggest a distant evolutionary relationship (Figure 1B). All twelve members of the complex were identified from mammalians to worms, except for the ACOT2 and STAR genes, which were missing from insects because of either their distinct protein architectural structure (there is a STAR-like sequence encoding for a much larger protein) and/or too much sequence divergence. Whether these members are orthologous or paralogous or even from distinct lineages remains to be evaluated by phylogenetic and evolutionary analyses. Thus, the top 500 hit sequences were retrieved from GenBank (http://www.ncbi.nlm.nih.gov/genbank) and subjected to further detailed phylogenetic and functional phylogenomic analysis.

### No functional divergence was detected between TSPOs in vertebrates and TSPOs prior to the gene duplication event

**Figure 2:**
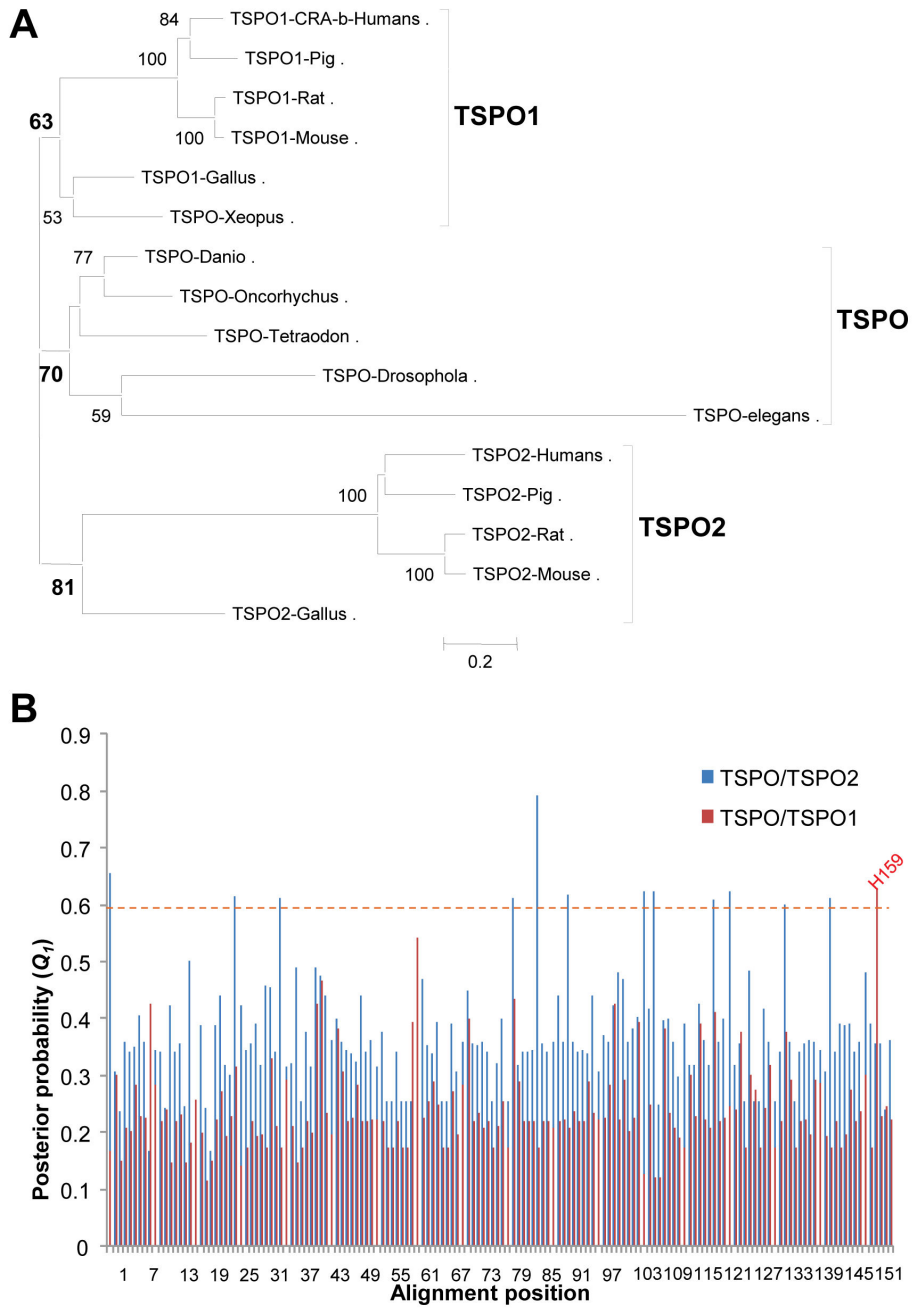
Functional divergence analysis of TSPOs before and after gene duplication. A. NJ-tree of TSPOs from human to worm. The evolutionary history was inferred using the Neighbor-Joining method [97]. The percentage of replicate trees in which the associated taxa clustered together in the bootstrap test (1000 replicates) is shown next to the branches. The tree is drawn to scale with branch lengths in the same units as those of the evolutionary distances used to infer the phylogenetic tree. The evolutionary distances were computed using the Dayhoff matrix-based method and are given in the units of the number of amino acid substitutions per site. All positions containing alignment gaps and missing data were eliminated only in pairwise sequence comparisons (pairwise deletion option). A total of 187 positions were analyzed in the final dataset. Phylogenetic analyses were conducted in MEGA4 [27]. The following sequences with accession numbers were used for the analysis: TSPO-Danio (NP_001006032), TSPO-Oncorhychus (AAK31586), TSPO-Tetradon (CAG06923), TSPO-Xeopus (AAH41505), TSPO-Drosophola (NP_608531), TSPO-*elegans* (NP_001129759), TSPO1-Pig (NP_998918), TSPO1-Mouse (P50637), TSPO1-Rat (NP_036647 NP_036647), TSPO1-CRA-b-Humans (NM_000714), TSPO1-Gallus (XP_416451), TSPO2-Gallus (XP_418037), TSPO2-Rat (XP_001063305), TSPO2-Mouse (NP_081568), TSPO2-Humans (NP_001010873), TSPO2-Pig (N166970). B. Site-specific profile for predicting critical amino acid residues responsible for type-I functional divergence between clusters TSPO (from invertebrates) and TSPO1 or TSPO2 as measured by posterior probability (*Q*1). Only one site, H159 (hTSPO1), is over 0.6 of *Q*1 value between TSPO/TSPO1, whereas there are 11 sites over 0.6 of *Q*1 value between TSPO/TSPO2.

TSPO, which is a high affinity cholesterol binding protein in the outer mitochondrial membrane and the central member of the steroidogenesis machinery, is encoded by a family of evolutionarily conserved genes with a variety of functions in Bacteria, Archaea, and Eukarya domains [23]. We previously reported that a new member of the TSPO gene family, *TSPO2*, exists in mammals and birds, plays a role in the re-distribution of intracellular cholesterol, and may be essential during erythrocyte maturation [38]. To determine which gene cluster evolved from genes prior to the gene duplication, we examined site-specific rate shifts to predict functional divergence (Figure 1C) [30,39]. We selected a total of 16 members of the TSPO protein family with origins in mammals, birds, fish, and invertebrates for this analysis (Figure 2). After sequence alignment and generation of the NJ tree, the data were loaded as an input to calculate the coefficients of functional divergence (*θ*) as an estimation of the functional divergence between *TSPO* genes before and after gene duplication. No significant functional divergence was detected between the clade TSPO1 (most vertebrates) and TSPO (fishes and invertebrates), whereas a significant difference was found between TSPO2 and TSPO1 as well as between TSPO2 and TSPOs, indicating that altered selective constraints on these residues prevent functional divergence but this is not the case for TSPO1/TSPOs (Table 1 and Figure 2B). These results suggest that invertebrate TSPOs likely possess the functions of mammalian TSPOs, despite an absence of relevant reports. In agreement with this observation, mammalian TSPO has been shown to successfully replace its bacterial ortholog in *Rhodobacter sphaeroides* [40].

**Table 1:** Functional divergence analysis of complex members in animals.

**Gene family**	**Comparison^a^**	***θ*^b^**	**SE^c^(θ)**	**LRT^d^**	***p*(LRT)^e^**
**TSPO**	TSPO1 *vs.* TSPO	0.2416	0.1722	1.9695	**0.1605**
	TSPO2 *vs.* TSPO	0.3727	0.1702	4.7951	0.0285
	TSPO1 *vs.* TSPO2	0.3976	0.1381	8.2926	0.0040
**STAR**	STAR *vs.* STAR-START1	0.4600	0.1518	9.1820	0.0024
	STARD3 *vs.* STAR-START1	0.2008	0.3988	0.2535	**0.6146**
	STAR *vs.* STARD3	0.6312	0.1262	25.0345	<0.0001
**ACBD3 (PAP7)**	ACBD3-v *vs.* ACBD3-iv	0.1144	0.0816	1.9655	**0.1609**
**ACBD1 (DBI)**	ACBD1-v *vs.* ACBD1-iv	0.2336	0.2225	1.1021	**0.2938**
	ACBD7 *vs.* ACBD1-iv	0.2520	0.1979	1.6212	**0.2029**
	ACBD1-v *vs.* ACBD7	0.7184	0.2767	6.7420	0.0094
**PRKAR1A**	PRKAR1A *vs.* PRKAR1	0.0650	0.1636	0.1579	**0.6911**
	PRKAR1B *vs.* PRKAR1	0.3696	0.1083	11.6384	0.0006
	PRKAR1A *vs.* PRKAR1B	0.3272	0.1274	6.5939	0.0102
**CYP11A1**	CYP11A1 *vs.* CytoCYPS	0.4258	0.0575	54.8458	<0.0001
	CYP11B1 *vs.* CytoCYPS	0.5520	0.0582	89.9629	<0.0001
	CYP11A1 *vs.* CYP11B1	0.2952	0.0439	45.1495	<0.0001
**VDAC**	VDAC1 *vs.* VDAC	0.1904	0.1389	1.8783	**0.1705**
	VDAV2 *vs.* VDAC	0.0674	0.1214	0.3080	**0.5789**
	VDAC3 *vs.* VDAC	0.0896	0.0801	1.2513	**0.2633**
	VDAC1 *vs.* VDAV2	0.1864	0.1154	2.6113	0.1061
	VDAC1 *vs.* VDAC3	0.3128	0.0938	11.1225	0.0009
	VDAV2 *vs.* VDAC3	0.2464	0.1196	4.2417	0.0394
**ANT**	ANT1 *vs.* ANT-is	0.3496	0.1131	9.5475	0.002
	ANT2 *vs.* ANT-is	0.9408	0.2812	11.1898	0.0008
	ANT3 *vs.* ANT-is	0.3800	0.1298	8.5668	0.0034
	ANT4 *vs.* ANT-is	0.2584	0.1053	6.0207	0.0141
	ANT1 *vs.* ANT-wm	0.4304	0.1017	17.9234	<0.0001
	ANT2 *vs.* ANT-wm	0.9992	0.1290	60.0214	<0.0001
	ANT3 *vs.* ANT-wm	0.0218	0.1896	0.0132	**0.9084**
	ANT4 *vs.* ANT-wm	0.0010	0.0224	0.0000	**1**
**ATAD3**	ATAD3 *vs.* ATAD3-is	0.0062	0.0775	0.0033	**0.9542**
	ATAD3 *vs.* ATAD3-wm	0.1028	0.0753	1.0260	**0.3110**
**ERK1/2 (MAPK3)**	MAPK3 *vs.* MAPK-is	0.0450	0.0866	0.2702	**0.6032**
	MAPK3 *vs.* MAPK-worm	0.1296	0.0884	2.1505	**0.1425**
	MAPK1 *vs.* MAPK-is	0.1088	0.1471	0.5468	**0.4596**
	MAPK1 *vs.* MAPK-wm	0.0010	0.0224	0	**1**
	MAPK3 *vs.* MAPK1	0.0522	0.1573	0.1101	**0.74**
**ACSL4**	ACSL4 *vs.* ACSL	0.2184	0.0338	41.8015	<0.0001
	ACSL3 *vs.* ACSL	0.1296	0.0368	12.3758	0.0004
	ACSL4 *vs.* ACSL3	0.1624	0.0331	24.0750	<0.0001
**ACOT2/3**	ACOT2/3 *vs.* ACOT	0.3088	0.1077	8.21727	0.0041
	ACOT4 *vs.* ACOT	0.3992	0.1209	10.8959	0.001
	ACOT6 *vs.* ACOT	0.1592	0.1635	0.94831	**0.3301**
	BAAT *vs.* ACOT	0.4528	0.0625	52.5212	<0.0001

a The abbreviations represent each category of the gene family, where the – v represents proteins with an origin in vertebrates, – iv represents proteins with an origin in invertebrates, – is represents proteins with an origin in insects, and – wm represents proteins with an origin in worms.

b The coefficient of functional divergence type I (θ) was calculated by maximum likelihood.

c SE, standard error.

d The likelihood ratio test (LRT) to compare the likelihood of the hypothesis indicating no functional divergence to the hypothesis assuming functional divergence.

e The *p*-value was estimated from the LRT, which approaches a χ^2^ distribution with 1 degree of freedom. The bold and/or underlined values indicate no significant difference during the evolution of the paired categories of proteins.

### STAR, ACBD1, and ACBD3, which are involved in cholesterol delivery to TSPO and TSPO activation, appear at the same time as the CRAC domain of TSPOs

**Figure 3:**
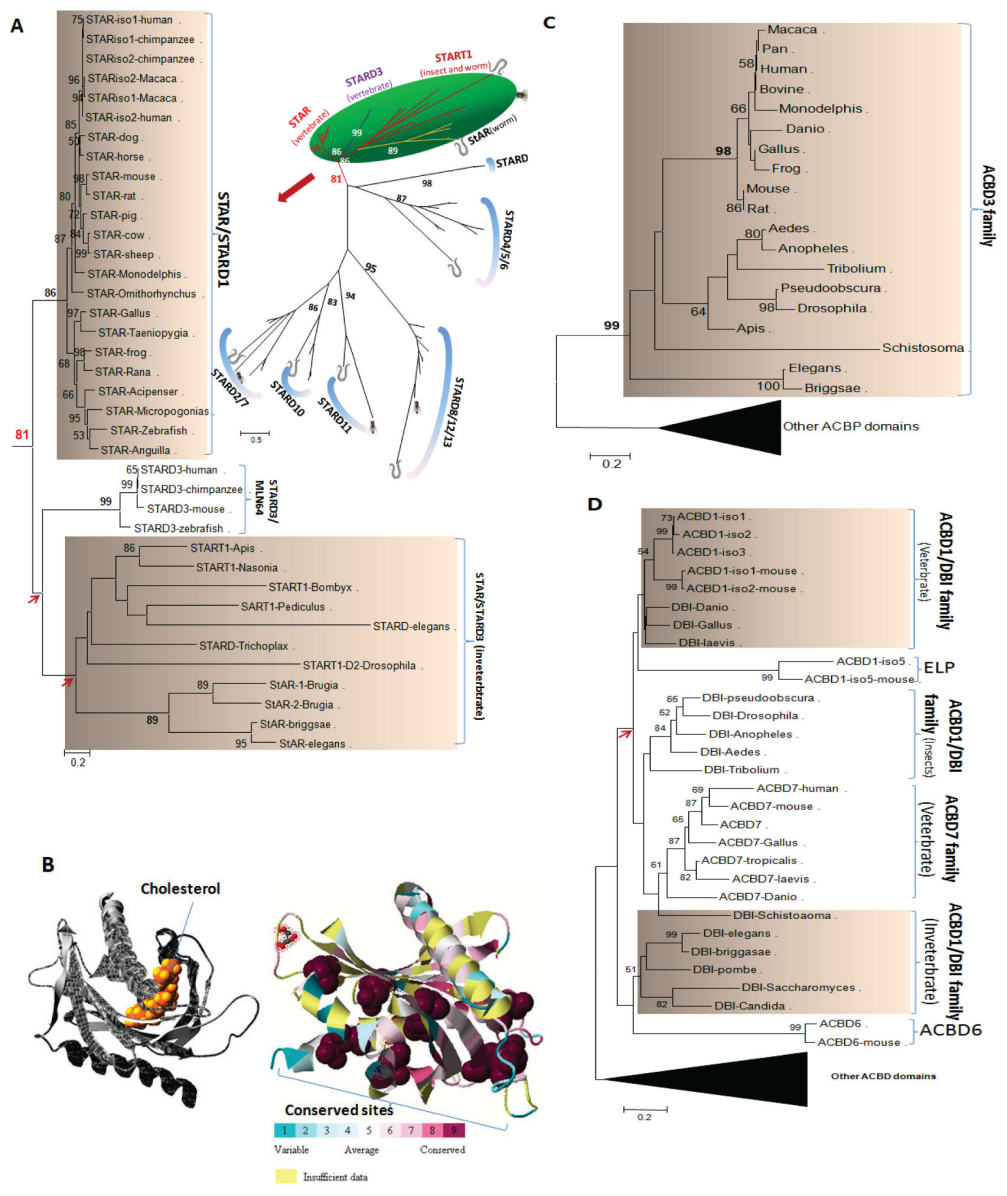
Conserved elements in steroidogenesis machinery involved in cholesterol transport from humans to worms. A. NJ tree for the START family. The orthologous genes of human STAR can be traced back to *C. elegans*. B. Homology modeling of the mouse STAR (left) and the evolutionary conservation profiles of the START family (right). The evolutionary conservation profiles are mapped on the homology 3-D ribbon model of mouse STAR, where the most conserved (score 9) residues are displayed by space-filled atoms. The amino acid residues are colored according to their conservation grades using the color-coding bar, with turquoise through maroon indicating variable through conserved, respectively [33]. C. NJ tree for the ACBD3 (PAP7) family. The orthologous genes of human *ACBD3* form a unique branch and are highly conserved within animals (adapted from ACBD3 review [4]). D. NJ tree for the ACBD1/DBI family. The orthologous genes of human ACBD1 are limited within vertebrates but exhibit various sequence divergences and gene duplications (e.g. ACBD7).

Although the TSPO proteins were found in almost every organism, the cholesterol binding domain (the CRAC domain, L/V/I-(X)1–5-Y-(X)1–5-R/K) at the C-termini of TSPO proteins mainly exists in members of the animal kingdom (Figure S1) [23,41]; however, the CRAC domain was found to be sparsely distributed in some bacteria and archaea, including the marine bacterium *Fulvimarina pelagi* and the thermophilic obligately-aceticlastic methane-producing archaeon *Methanosaeta thermophila*. Whether the CRAC domains within prokaryotic organisms are the evolutionary source of the animal CRAC domain or whether these domains evolved later from accumulation of random neutral changes and natural selection remains to be established. Nevertheless, our phylogenetic analysis indicates that the cholesterol and lipid binding protein STAR/STARD1 and the acyl-CoA binding proteins ACBD3 (PAP7) and ACBD1 (DBI), which all interact with TSPO and function at the outer mitochondrial membrane, co-appeared within the animal phylum at the same time as the CRAC domain (Figure 3).

STAR proteins comprise a highly conserved protein family within the vertebrates, and these proteins function with TSPOs in steroidogenesis in a concerted manner [3,5–7,42]. Phylogenetic analysis revealed that STAR is part of a group of STARD-containing proteins, including STARD3/MLN64, insect STAR-related lipid transfer 1 (START1), and worm STAR and STARD3 with strong bootstrap support (Figure 3A). This observation is consistent with previous reports that the STAR-domain in STARD3 may share the same function as STARD1/STAR proteins and, noticeably, STAR and STARD3 form one of six conserved subfamilies of the STARD-containing proteins [43,44]. In general, all the START domain-containing proteins are found in vertebrates, insects, and worms. For STARD1/STARD3, worms have a shorter form of a START domain-containing protein, and this protein is likely the ancient form of the vertebrate STAR, whereas START1 from worms and insects is the ortholog of vertebrate STARD3 (Figure 3A). Functional divergence analysis, however, showed that vertebrate STAR exhibited different functional constraints during evolutionary history, indicating that worm STAR may not have the same function as the ones in vertebrates, whereas the orthologs of STARD3 had no detected functional divergence (Table 1). On the other hand, the three-dimensional homology model demonstrated that the main conserved sites are responsible for the maintenance of its structure, which has been essential for its molten globule conformational change upon binding cholesterol via a hydrogen bond (Figure 3B) [45]. This finding suggests that cholesterol binding by this family of proteins occurs within a conserved hydrophobic binding tunnel or groove that can accommodate the molecule As previously reported this tunnel/groove is formed by strands β5 and β6 (residues 335–344), strands β2 and β3 (residues 277–279), and the region around the N-terminal end of the C-terminal α-helix (residues 410–425) of the START domains of MLN64 or STARD3, even though binding is not strictly related to the specific binding sites that consist of conserved amino acids [31,46].

ACBD3, previously known as peripheral-type benzodiazepine receptor and cAMP-dependent protein kinase-associated protein 7 (PAP7) or Golgi complex-associated protein of 60kDa (GCP60), is a Golgi-resident protein and is involved in Golgi body-mediated signaling during mitosis [4]. ACBD3 is rapidly released from the Golgi upon hormonal stimulation in steroidogenic cells and translocates to the mitochondria. ACBD3 functions as an A-kinase anchor protein that binds and brings the regulatory subunit Iα of the cAMP-dependent protein kinase to TSPO [8]. This interaction allows phosphorylation of STAR, which is also targeted to mitochondria thought its N-terminal mitochondrial targeting sequence [1]. The phylogeny of the ACBD3 family of proteins can be traced to worms and insects; the family forms a significantly different branch from any other ACBD domain-containing protein (Figure 3C). Notably, ACBD3 appears to be distributed only within animals, and no functional differentiation of ACBD3s was detected between vertebrates and invertebrates (Table 1).

ACBD1, also known as DBI, is one of several other ACBP domain-containing proteins [4,47]. ACBD1 was previously known as endozepine, a peptide cleaved by endo- and exo-peptidases to form bioactive processing products [48,49]. The interaction between ACBD1 and TSPO is thought to be critical for the acute stimulation of steroidogenesis by hormones, and ACBD1 serves as an endogenous TSPO ligand [50–52]. Phylogenetic analysis has shown that ACBD1 from vertebrates, invertebrates, and fungal yeasts form a branch that is different from other ACBP domain-containing proteins. Exceptions include ACBD6, which is an ankyrin repeat-containing protein and may play a role in hematopoiesis and blood vessel development [53], and ACBD7, which is distributed in a limited number of tissues, such as brain, and is therefore also known as brain ACBP (B-ACBP; Figure 3D) [54]. For these ACBDs, no difference was detected in functional divergence within animals (Table 1).

Taken together, these results indicate that the co-appearance of the CRAC domains of TSPOs with STAR, ACBD3, and ACBD1 occurred as early as *C. elegans* with some functional conservation in terms of cholesterol metabolism in steroidogenesis.

### PRKAR1A and mitochondrial CYP11A1 are the result of gene duplication events in vertebrates

**Figure 4:**
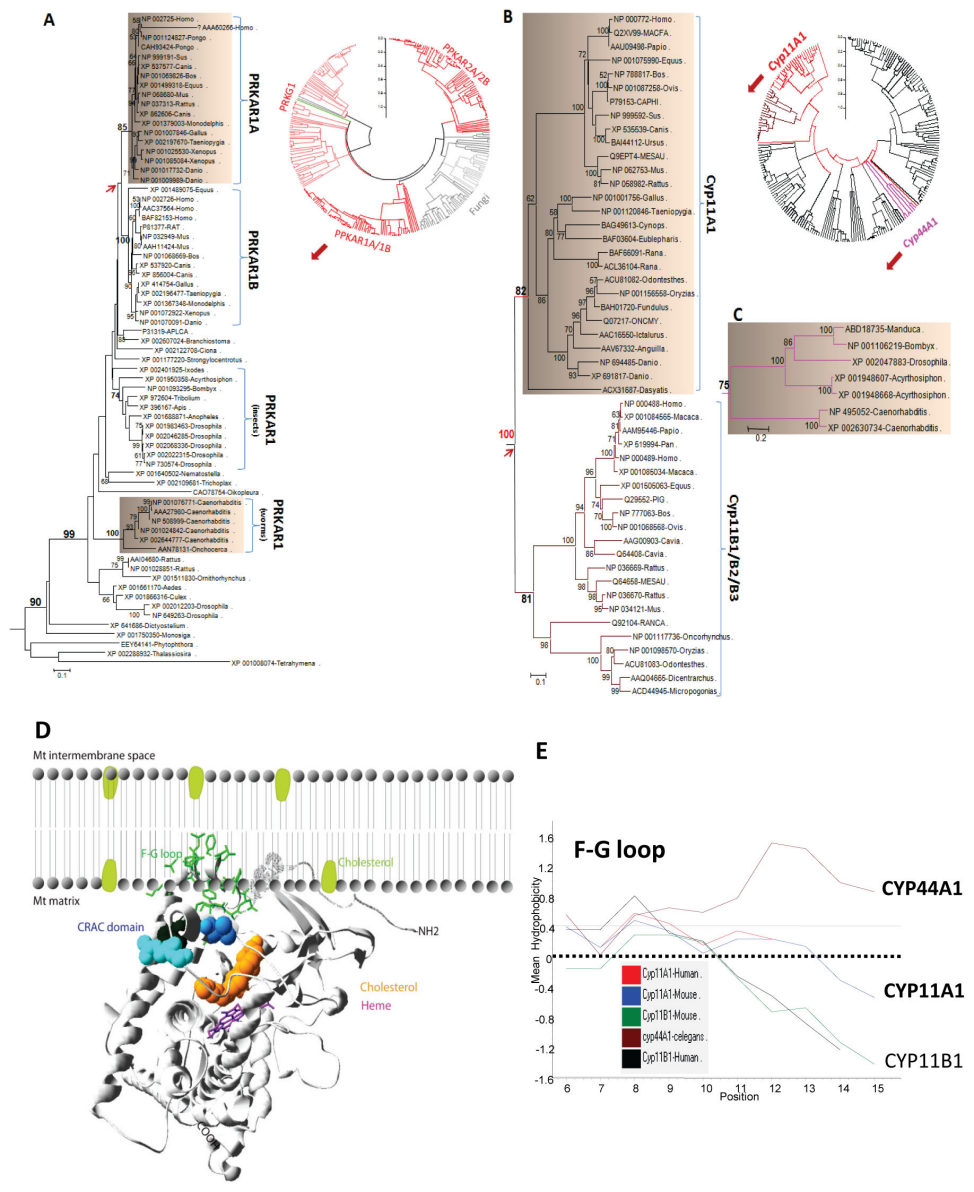
Conserved elements of the steroidogenesis machinery involved in cholesterol transport in vertebrates. A. NJ tree for the PPKAR1A family. The orthologous genes of human *PPKAR1A* are distributed within entire animals with strong bootstrap support, although gene duplication events occurred during vertebrate evolution. B. NJ tree for the CYP11A1 family. The orthologous genes of human *Cyp11A1* are limited within vertebrates and were duplicated from Cyp11B1/B2/B3. C. The phylogeny of the invertebrate Mt CYP enzymes, CYP44A1 (NP_495052) and CYP314A1 (XP_002047883). D. Illustration of insertion of the human CYP11A1 into the mitochondrial inner membrane via an F-G loop, which is attached to the mitochondria inner membrane and is highlighted via a stick structure. The CRAC domain is shown as a ball-and-stick representation, and the cholesterol molecule is sitting within the steroid binding pocket and depicted in brown. The model was derived from PDB code 3NA1 (color in grey). E. Hydrophobicity profile of the F-G loops from CYP11A1, CYP11B1, and CYP44A1. The hydropathy indices were determined as described by Kyte & Doolittle [98]. Hydrophobic residues are indicated by positive values.

The cAMP-dependent protein PRKAR1A is a critical component of hormone-induced steroid biosynthesis [3,55]. Phylogenetic analysis showed that PRKAR1A from all animals formed a branch with strong bootstrap support (Figure 4A). PRKAR1B in the branch was apparently generated from a gene duplication event in vertebrates. PRKAR1B appears to occur with a tissue-specific isoform small diameter tropomyosin receptor kinase (trkA) not only in the nervous system in receptor-positive dorsal root ganglion cells [56] but also in germ cell stages with a differential expression pattern [57]. In addition, the functional divergence between the PRKAR1A and PRKAR1B families is significant, despite a lack of obvious difference between the vertebrate PRKAR1A and the invertebrate form (Table 1). These results suggest that invertebrates have a PRKAR1A protein with the same or similar function as the one in vertebrates.

**Figure 5:**
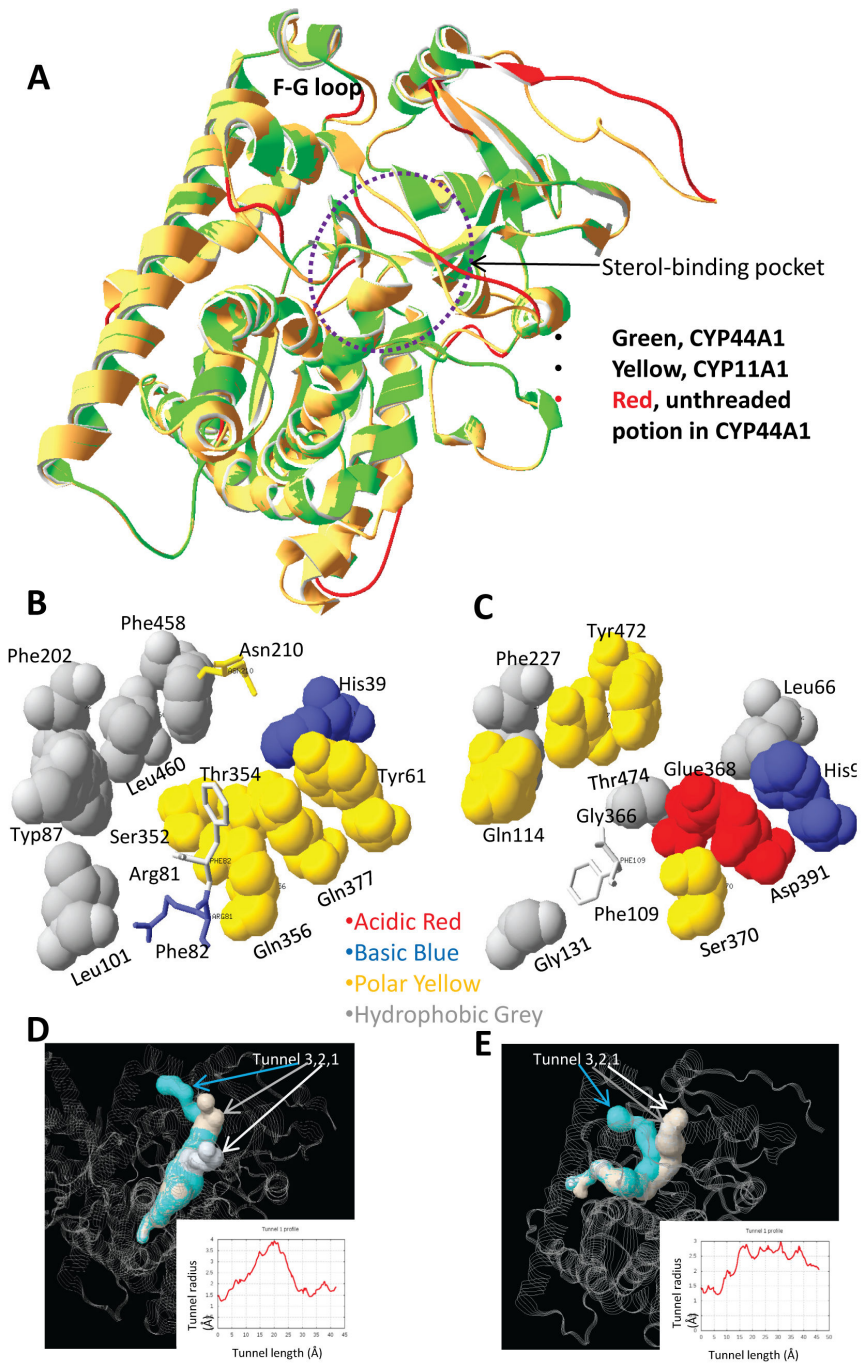
Comparison of putative steroid binding pockets from *C. elegans* CYP44A1 and CYP11A1. A. Superimposition of the *C. elegans* CYP44A1 threading model (green) with human CYP11A1 (yellow). The F-G loop, which attaches to the mitochondrial inner membrane, and putative steroid binding pocket are indicated. B. The sites responsible for steroid binding in human CYP11A1 (PDB, 3NA1). C. The sites corresponding to the human CYP11A1 steroid binding pocket in Figure 6B and S2. The colors of each site are as illustrated. The unmatched sites are shown in the stick structure, and the remainder of the sites is shown in the space-filling model. D. Three predicted channels involved in HEM and steroid binding in CYP11A1, which is in complex with adrenodoxin reductase (AdR). E. Three potential channels involved in steroid binding in CYP44A1. The channels were predicted using MOLE 2.0 online service (http://mole.upol.cz) [99].

**Figure 6:**
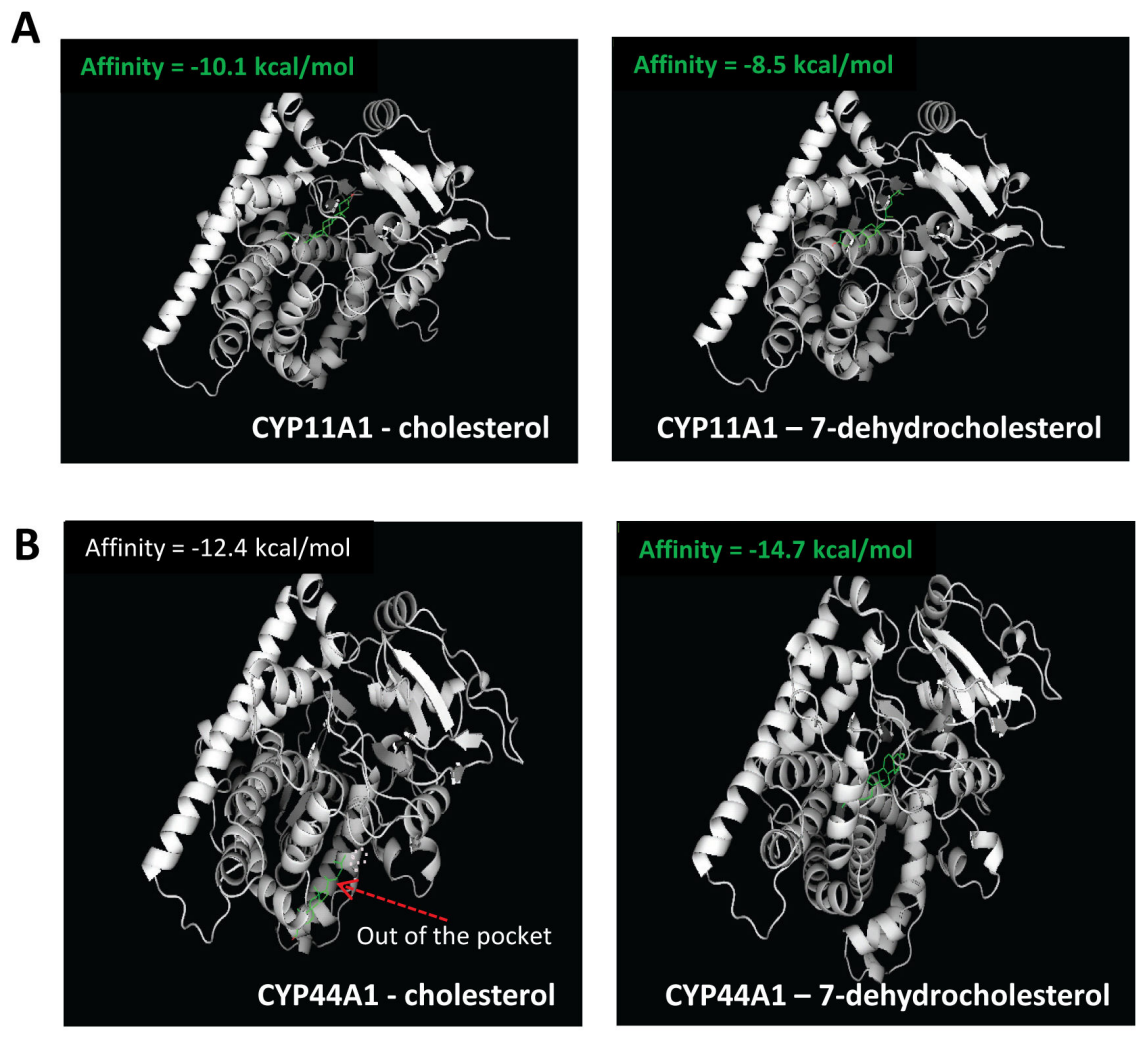
Comparative molecular docking analysis with the homology models of *C. elegans* CYP44A1 and the crystal structure of human CYP11A1 docking with cholesterol and 7-dehydrocholesterol. A. Docking of CYP11A1 with cholesterol (left) and 7-dehydrocholesterol (right). B. Docking of CYP44A1 with cholesterol (left) and 7-dehydrocholesterol (right). The docking results are shown as the top conformation in rank of free energy. Protein structures are shown in ribbon representation. Cholesterol is in a ball-and-stick model (green).

CYP11A1 is the only mitochondrial cytochrome P450 enzyme to convert cholesterol to pregnenolone [58]. Similar mitochondria-type cytochrome P450 enzymes have been found in *C. elegans* and in insects, even though the unique mitochondria CYP44A1 enzyme in *C. elegans* has not been strongly associated with endocrinology or development of the nematode [59]. One of the two types of mitochondrial cytochrome P450 enzymes (such as CYP302, CYP314, CYP315) in insects plays a role in essential physiological functions, while another enzyme (such as CYP12A1) is involved in xenobiotic metabolism [60,61]. Although the latter enzyme has not been directly associated with insect ecdysteroid formation, this protein has been proposed to have an ancestral role in steroidogenesis [60,62]. Phylogenic analysis demonstrated that CYP11A1 and CYP11B1 form a branch within all vertebrates and likely arose from a gene duplication event during evolution (Figure 4B). Mitochondrial cytochrome P450 enzymes formed a different branch from the rest of the cytochrome P450 enzymes with a specific mitochondrial cytochrome P450 enzyme motif (C-GRR—E) that interacts with the redox partner adrenodoxin and exhibits enzymatic activity (Figure 4C and S2). Typical features of CYP11A1 in relation to its mitochondrial function include possession of an F-G loop targeting the mitochondrial inner membrane, a putative CRAC domain adjacent to the membrane, and a hydrophobic steroid binding pocket/channel (Figure 4D). Functional divergence analysis suggested that the two mitochondrial enzymes CYP11A1 and CYP11B1 are totally dissimilar and that both of these enzymes are different from that in invertebrates (Table 1). Additionally, several vertebrate sequence features, such as the presence of the F-G loop, conserved alpha-helix structure, cholesterol access channel/recognition sites for substrate entry, and substrate binding within the CYP11A1 family of proteins, are variable in cytochrome P450 enzymes from worms (Figure 4E and S2) [35,63]. These findings support the previous suggestion of a rapid evolution of cytochrome P450 enzymes [64]. The common feature of CYP11A1 and CYP11B1 is that both enzymes attach to the inner mitochondrial membrane [65]. From hydropathy analysis, it is clear that the worm CYP44A1 is more hydrophobic; therefore, this enzyme is also expected to be membrane associated (Figure 4E) and possess an intramolecular tunnel to embrace a steroid/ heme molecule, as the cholesterol molecule is 16.7 Å in length and 2.5-3.6 Å in width (Figure 5). Indeed, we found that CYP44A1 and CYP11A1 have similar binding affinity to 7-hydrocholesterol. Comparative ligand docking analysis using the homology model of CYP44A1 indicated that this enzyme favors the binding of 7-hydrocholesterol over cholesterol in comparison to human CYP11A1 (Figure 6). These results suggest that 7-dehydrocholesterol binding by mitochondrial cytochrome P450 enzymes originated at the nematode and evolved to favor cholesterol binding in humans. Since the mitochondrial cytochrome P450 enzymes are seemingly present only within the animal kingdom, these enzymes likely share a similar endocrine function.

### Mitochondrial VDAC1 and ANT1 are members of multiple gene families in vertebrates, but ATAD3 only occurs within primates

**Figure 7:**
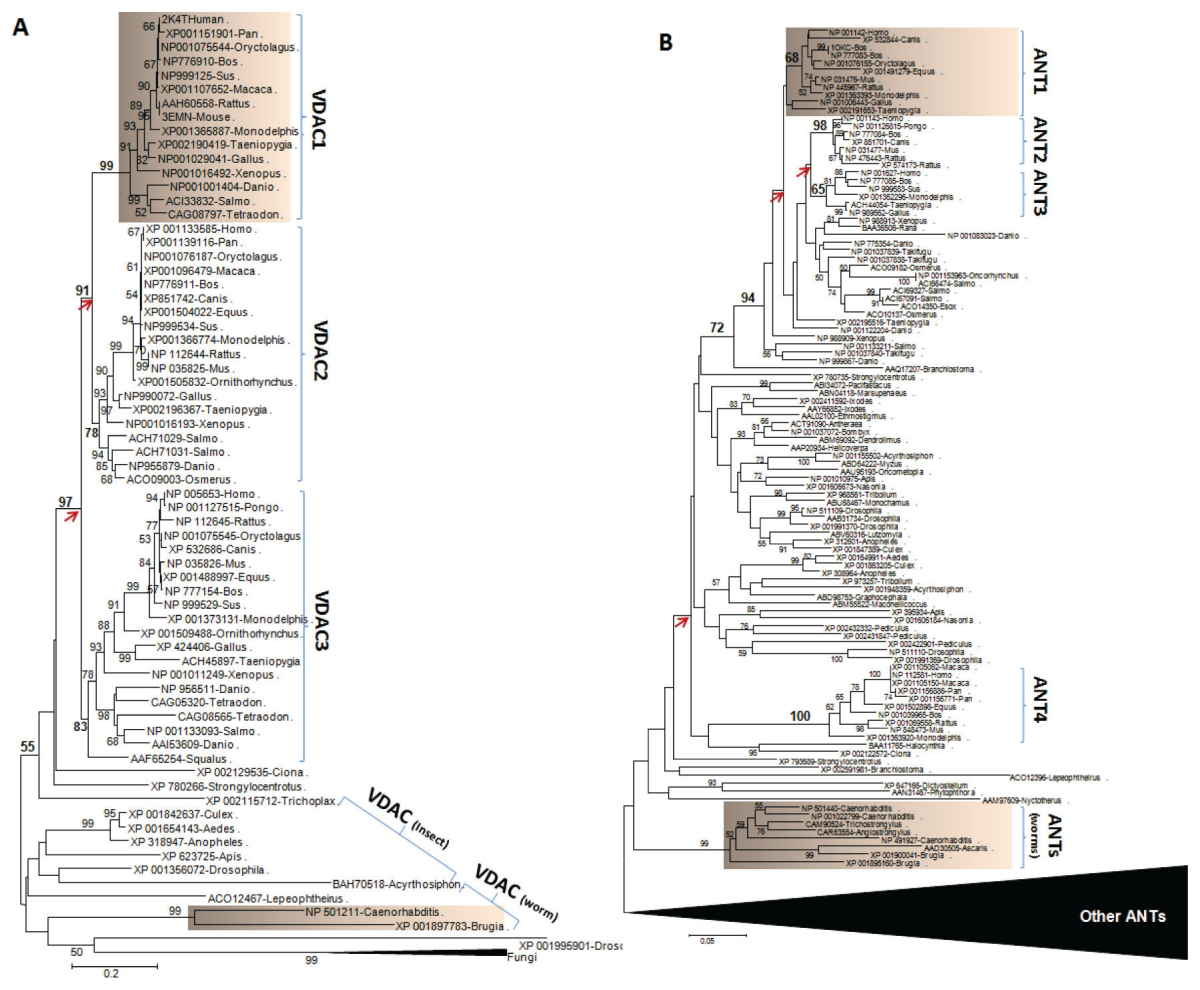
Phylogenetic analysis of animal VDAC and adenine nucleotide translocase (ANT) families. A. The phylogenetic tree of animal VDACs is based on the NJ analysis of entire animal sequences retrieved from GenBank. Fungi sequences were collapsed in the tree. B. The phylogenetic analysis of ANTs is based on the NJ analysis of entire animal sequences retrieved from GenBank. The ANT sequences other than animals are collapsed in the figure. The obvious gene duplication events are indicated by arrows. Levels of confidence of the nodes are only provided if support is greater than 50% by bootstrap analysis.

VDAC1 and ANT1 are considered the base of the complex on the mitochondrial surface, and previous data showed that phosphorylated STAR interacts with VDAC1 and that TSPO is associated with VDAC and ANT for steroidogenesis [12,66,67]. Although the function of VDAC in steroidogenesis is known [7,68], the role of ANT in this process has recently been questioned [7]. In vertebrates, multiple gene families of VDACs (VDAC1-VDAC3) and ANTs (ANT1-ANT4) formed during evolution (Figure 7). Although different paralogous genes exhibit tissue-specific protein distribution, VDAC1 and ANT1 are the dominant factors from these gene families involved in mitochondrial function and steroidogenesis [7,12,66,68]. In invertebrates, only one VDAC and one ANT gene have been identified. Functional divergence analysis indicated that there was no significant difference between vertebrate VDAC1 and invertebrate VDAC, while considerable divergence was observed among ANT1 to ANT4 from vertebrates and invertebrates (Table 1), except that only ANT3 and ANT4 have no detectable difference in functions between vertebrates and worms. This divergence may have resulted from rapid evolution since the duplication events occurred before the divergence of avians and mammals, whereas gene duplication events of VDACs occurred before the appearance of vertebrates. Taken together, these data suggest that one of the machinery members, VDAC, which is located on the outer mitochondrial membrane and closely interacts with TSPO and STAR [7,68], is conserved within animals.

**Figure 8:**
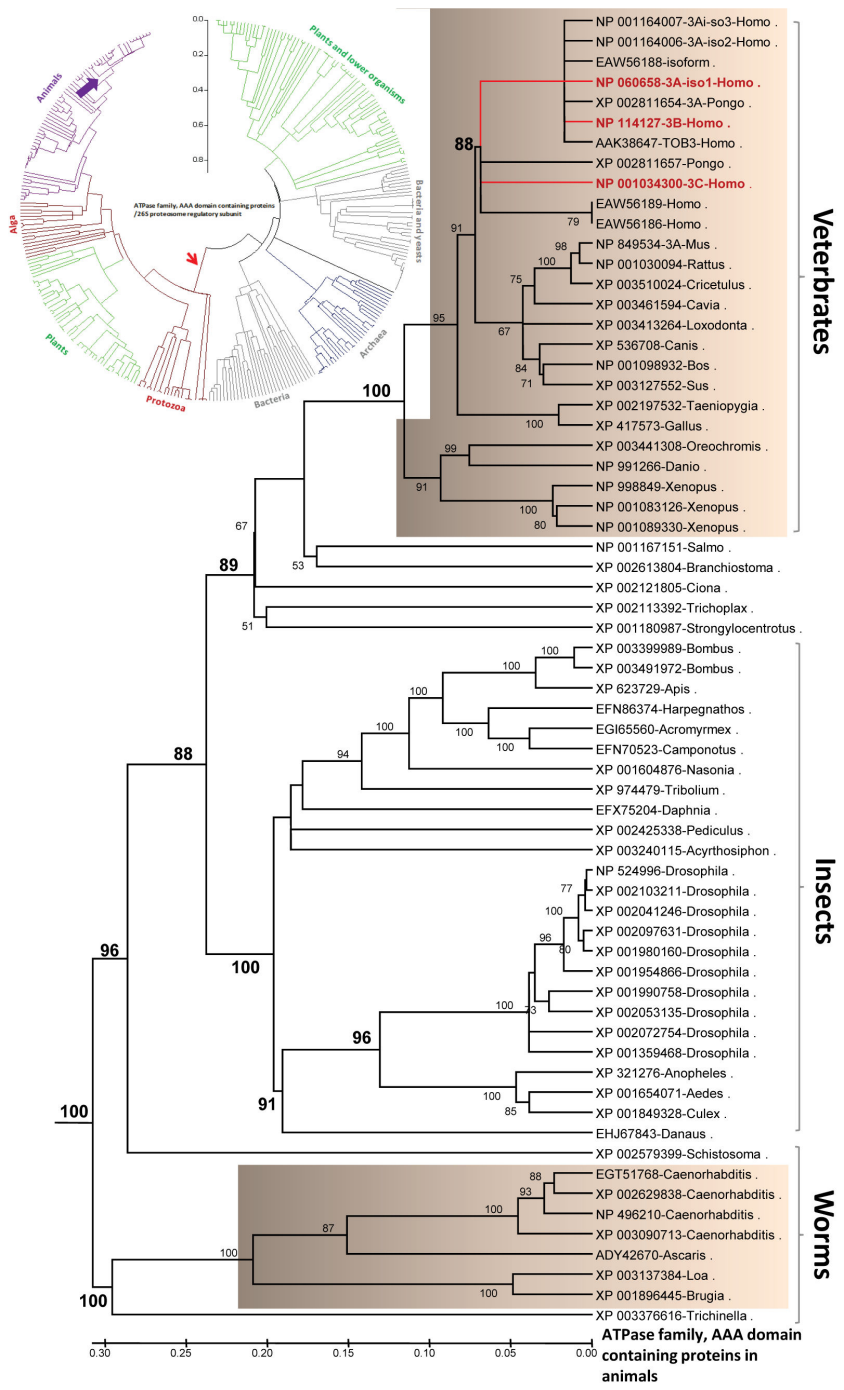
Phylogenetic analysis of ATAD3 sequences. The top 500 sequences related to the mammalian ATAD3 were used in this analysis as shown as a circle phylogenetic tree (inset). This analysis illustrated the evolutionary distribution of the orthologous ATAD3 genes throughout animals, plants, and protists (such as alga and protozoa), indicated by a red arrow. Animal ATAD3-related sequences were subtracted as shown in an NJ tree. ATAD3 genes from vertebrates, insects, and worms clustered as a phylogenetic branch with strong bootstrap support. Gene duplication events in humans and likely in other primates as well are highlighted by red bold letters as 3A, 3B, and 3C after each GenBank accession number. The bootstrap values greater than 50% are indicated.

ATAD3A is preferentially located at the outer-inner mitochondrial membrane contact sites and has been recently shown to be an integral part of the cholesterol transport machinery for steroidogenesis [7,69]. ATAD3 likely provides an extra layer of energy in this machinery as a result of its ATP-binding properties. ATAD3 is an evolutionarily conserved orthologous protein found in worms, insects, and humans (Figure 8) as well as in protists (e.g., algae and protozoa) [70]. No *Atad3* orthologous gene has been identified in yeast where the mitochondrial contact sites have been systematically studied [71]. In primates, the tandem arrangement and proximity of the ATAD3 genes have arisen by gene duplication, while the other organisms possess a single gene with a monophyletic origin with no functional divergence between vertebrates and invertebrates (Table 1).

### Conserved signal transduction factors including MAPK3 and variability of ASL4 and ACOT6 in vertebrates suggests unique vertebrate signaling features distinct to invertebrates

MAPK3, also known as ERK1/2, is a member of the mitogen-activated protein kinase (MAPK) family and is involved in the regulatory pathway of mitochondrial cholesterol transport during steroidogenesis [10,11]. All members of the MAPK3 and MAPK1 families from all animals examined formed one phylogenetic branch with strong bootstrap support (Figure S3). Functional divergence analysis indicated a lack of a significant difference between the two subfamilies (Table 1), suggesting that the mechanism of action of this family of kinases is likely highly conserved.

Two other members in the complex, ACS4 and ACOT2/3, are essential for arachidonic acid release from phospholipids and activation of STAR transcription in steroidogenic cells [11,72–74]. Phylogenetic analysis showed that ACSL3 and ACSL4 formed a branch with ACSL from worms and insects with strong bootstrap support (Figure S4); however, the members from these two subfamilies are significantly different from each other (Table 1), suggesting that the ACSL isoforms have different preferences for fatty acid release as evident by the markedly difference preferences of ACSL4 and ACSL3 for AA, docosahexaenoic acid, and eicosapentaenoic acid [75]. The gene families encompassing ACOT2/3 were actually found only in animals and bacteria (Figure S5). The complicated phylogeny in this gene family suggests that the available fatty acid sources have some species-specific features and/or that the genes are likely under positive selection. In conclusion, ACSL4 is relatively more conserved, whereas ACOT2/3 is quite variable with respect to its predicted functional divergence.

## Discussion

Steroid hormone biosynthesis is a well-orchestrated and tightly controlled metabolic pathway comprised of a series of successive enzymatic reactions catalyzed by cytochrome P450 and hydroxysteroid dehydrogenase enzymes located in mitochondria and the endoplasmic reticulum. In contrast to other biological systems where the enzyme levels can be limiting, substrate availability determines the rate of steroid formation and is the limiting factor in the process of steroidogenesis. This limitation is based on the fact that the substrate is cholesterol, a hydrophobic molecule residing in intracellular stores distant from mitochondria where steroidogenesis begins with the first metabolic enzyme CYP11A1. Steroid production in steroidogenic tissues occurs either in response to local needs as in the cases of placenta, brain, and thymus or in response to developmental, hormonal, or environmental stimuli as in the case of the adrenal glands and gonads, which synthesize large amounts of steroids to support adaptation and function of the entire body. These stimuli induce steroid biosynthesis by accelerating cholesterol transport into mitochondria in a timely and tightly controlled manner. Our current knowledge about cholesterol transport is based on extensive studies of complex members in mice and humans [2,3,5,11,76,77]. In comparison, the main precursor of steroid biosynthesis in insects and/or worms is 7-dehydrocholesterol, an entity that is translocated into mitochondria for conversion to the first intermediate metabolite(s) during steroid synthesis [78]. Whether transport machinery similar to that observed in mammals exists to assists the movement of this precursor into the mitochondria is unknown at this time, although this question is of great interest not only to endocrinologists but also to evolutionary biologists. The current study was undertaken to trace the evolutionary origin of the steroidogenesis machinery members involved in cholesterol transport among animals.

The machinery involved in cholesterol transport for steroidogenesis in mammals appears to be one of the most conserved biological systems. All twelve members examined have been monophyletic orthologs with no significant functional divergence. Our recent study on TSPO-related sequences revealed that the warm blooded animals, including mammals and avians, possess one additional gene in the TPSO family, TPSO2, which has a specialized function during erythropoiesis [38]. Whether the function of TSPOs in other vertebrates is similar to TSPO or to the newly described TSPO2 has not yet been reported. We hypothesize that the TSPO involved in steroidogenesis in most steroidogenic tissues, such as testis, ovary, brain, placenta, and adrenal gland, is inherited from the ancestral gene at least with regard to the sequence features [23]. Indeed, TSPO in zebrafish has been reported to play a key role in erythropoiesis in a fashion independent of the cholesterol binding site; however, the role of TSPO in steroidogenesis has not yet been fully explored, particularly for larval stages and later in the adult, for its implication during steroidogenesis [15]. All vertebrates likely share a similar mechanism of hormone-induced steroidogenesis with a rate-limiting step in cholesterol transport into mitochondria, despite the presence of gene duplication events [79].

Interestingly, the appearance of the TSPO CRAC domain and other core members of the machinery, including STAR, ACBD3/PAP7, ACBD1/DBI, PKAR1A, ATAD3, and VDAC1, occur at the same time within the entire animal kingdom. Except for STAR, the members have no functional divergence from worms/insects to humans. Previous reports indicated that mitochondrial cholesterol transport in steroidogenesis functions at a slower rate without STAR and that STAR is able to increase the rate of cholesterol trafficking [5]. Moreover, STAR-independent mechanisms have been proposed to explain the intracellular trafficking of cholesterol [80]. Additionally, in mammalian term placenta, no STAR activity is involved in the steroidogenesis, although mutation of this protein results in lipoid congenital adrenal hyperplasia but only after birth [81]. Although STAR has been found in brain [82], the regulation of neurosteroid formation by hormones and the presence of hormone-induced STAR in brain remain elusive. Evidence suggests that the function of STAR evolved later than the functions of the other members of the machinery, even though this factor is highly conserved within mammals.

**Figure 9:**
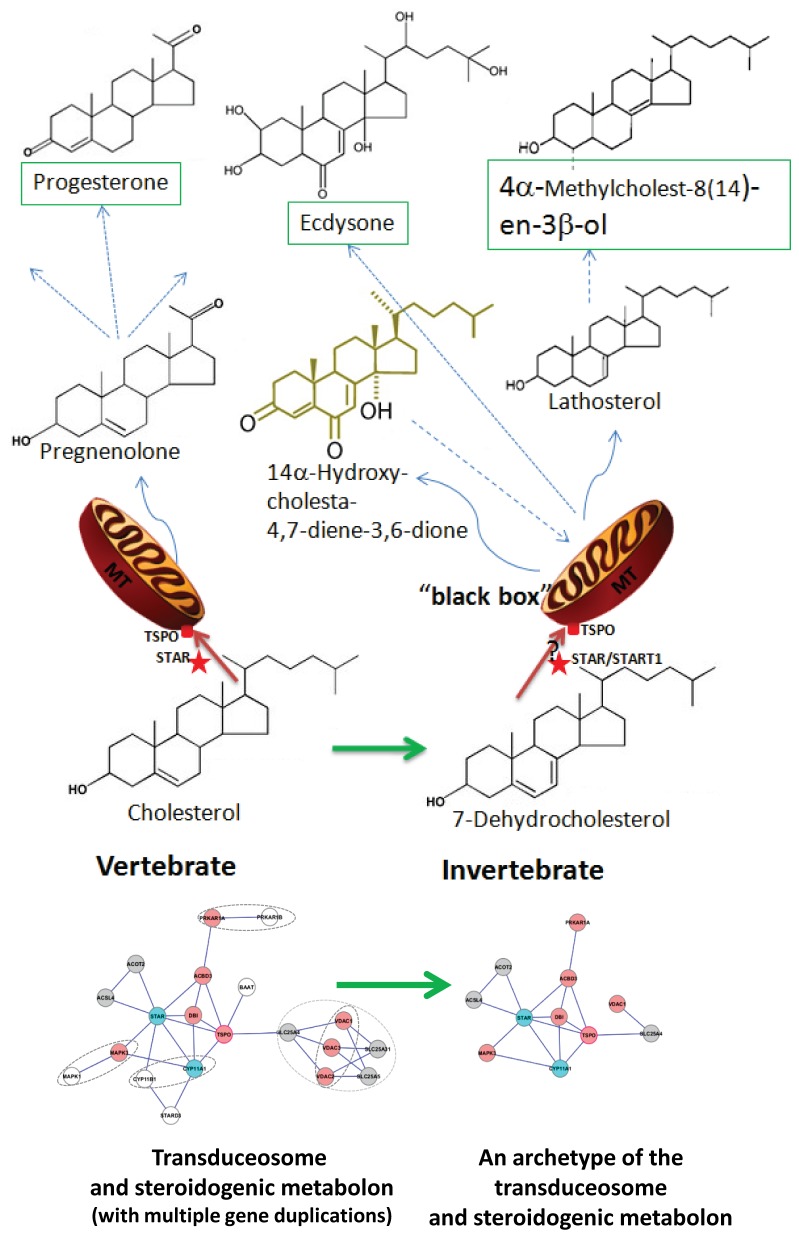
A diagram of the complex of evolutionary steroidogenesis in animals. The main players in the complex, TSPO and STAR, are indicated on the surface of mitochondria. Precursors (cholesterol for vertebrates and 7-dehydrocholesterol for invertebrates), and first metabolites (pregnenolone for human and lathosterol for worms), intermediate metabolites (progesterone), and/or final products (ecdysone for insects) are indicated. The “black box” refers to the initial steps of steroidogenesis in insects as the enzymes in this process are unknown [100]. The predicted functional interaction networks of the proteins involved in the complex was generated using version 9.0 of the STRING database [101]. The dotted oval circles indicate the gene duplication event. Red indicates the gene is conserved from mammals to invertebrates, light blue indicates genes with sequence divergence but with similar biological function, grey indicates genes with divergence in both sequence and function, and white indicates that the duplicated gene may not be involved in the complex.

Our systematic analysis on the phylogeny and functional divergence of the cholesterol transport machinery for steroidogenesis revealed that most members from mammals are evolutionarily conserved with monophyletic origin, whereas some members of the complex in vertebrates arose through a series of gene duplication events. None of the members of the machinery have significant functional divergence, suggesting a conserved general mechanism for steroidogenesis (Figure 9). All of the machinery members exist in worms and the insects examined, although some of these proteins, such as STAR, CYP11A1, ANT1, possess an inferred functional divergence, indicating the presence of some unique features in steroid biosynthesis in invertebrates. Actually, the first steroid product generated in mitochondria is 14α-hydroxy-cholesta-4,7-diene-3,6-dione in insects and lathosterol in worms, respectively [83,84]. Additionally, the precursor of steroid synthesis in invertebrates is 7-dehydrocholesterol instead of cholesterol [78]. Taken together, these results suggest that an archetype of cholesterol transport machinery for steroidogenesis exists within the whole animal kingdom.

We believe that the presence of this cholesterol transport machinery in invertebrates likely reflects this feature, assigned as an archetype of the prototypic mammalian cholesterol transport machinery for steroidogenesis established prior to the origins of vertebrates about 600 million years ago [85,86]. This archetype complex likely plays a role in metabolizing 7-dehydrocholesterol instead of cholesterol for both insects and worms. Indirect evidence in support of this notion comes from the finding that the steps between 7-dehydrocholesterol and the trideoxyecdysteroids occur in mitochondria [87]. Moreover, previous reports have shown that human CYP11A1 can actually process 7-dehydrocholesterol into 7-dehydropregnenolone [88,89], and our docking analysis indicated that the 7-dehydrocholesterol binding properties of CYP11A1 can be traced down to the nematode CYP44A1. Further experimental confirmation will shed a light on this “black box” of ecdysteroidogenesis in the prothoracicotropic hormone signal transduction pathway [90–92].

Variable outer layers of the conserved machinery exist in different organisms, including invertebrates and vertebrates. The steroid synthesis enzymes and a number of the members in the complex involved in signal transduction are functionally different between invertebrates and vertebrates. Such members are CYP11A1, which is responsible for the conversion of cholesterol to pregnenolone, ACSL4, which is essential for activation of STAR transcription, and ACOT2/3, which is involved in arachidonic acid release from phospholipids. The differences in the final steroids or in the intermediate metabolites may be attributable to the participating metabolic enzymes, such as CYP11A1. As in a previous report on the major enzymatic players including the steroidogenic cytochrome P450 enzyme superfamily implicated in steroidogenesis on the whole metazoan scale, three basal branching points of steroidogenesis exists in vertebrates, nematodes, and arthropods [21]. The similarities were observed in the variable signaling pathways that involve ANT1, ACSL4, and ACOT2/3. Notwithstanding, MAPK3 (ERK1/2) is highly conserved in all animals, and this characteristic may reflect another conserved biological system that functions *via* protein phosphorylation in the steroidogenic pathways in addition to protein kinase A signaling [76]. In addition, more and more proteins have been and/or will be identified to be involved in the early rate-limiting and regulatory steps of steroidogenesis. Indeed, the 14-3-3 protein family and mitochondria acetyl-CoA acetyltransferase (ACAT1) that converts two acetyl-CoAs into CoA, where the latter as an acyl group carrier is known for its role in transferring fatty acids from the cytoplasm to mitochondria for β-oxidation, were proposed to be involved in the origin of life on earth [93,94].

In conclusion, our studies revealed universal cholesterol transport machinery for steroidogenesis in vertebrates, where the complex is highly conserved in sequence within mammals and in function within all vertebrates, although some complex members suffered gene duplication events during their evolutionary history. Of interest, a similar mechanism may function in nematodes and arthropods, since an unveiled archetypical machinery exits in the animal kingdom and contains five core members of the complex, including TSPO and STAR in worms. STAR is likely not as active as its modern mammalian orthologs, suggesting that the function of STAR evolved later than TSPO and the other members. Other variable features of the machinery include cytochrome P450 catalytic enzymes involved in specific steroid biosynthesis and signal transduction proteins involved in the regulation of steroid biosynthesis for the transcriptional activation and phosphorylation of STAR.

The findings reported herein may help us distinguish an ancient function from evolving function(s) in steroid biosynthesis, metabolism and signaling as well as understand mechanisms responsible in conferring hormone-sensitivity of the steroidogenic ability of cells and tissues. For example, brain and placenta, the two hormone, and thus STAR-independent steroidogenic tissues seem to possess the ancient function to metabolize 7-dehydrocholesterol; the cholesterol/7-dehydrocholesterol ratio in brain increases during development, and 7- dehydrocholesterol in placenta is metabolized to biologically active 7-dehydropregnenolone [1,89,95,96]. Thus, the archetype of the cholesterol transport complex found in lower animals could be reflected in the hormone-independent placenta and brain steroid biosynthesis. Finally, advancing our understanding of the evolution of the first and critical step in steroidogenesis occurring at mitochondria may also unveil evolutionary aspects of mitochondria structure and function. The data presented reveals the presence of an universal cholesterol transport machinery in vertebrates. We believe that these findings constitute an advance in understanding the evolution of steroidogenesis, the origins of developmental and metabolic disorders associated with abnormal production of steroid hormones.

## Supporting Information

Figure S1 **Sequence alignment of TSPO CRAC domains.** Partial sequences, including transmembrane 5 (TM5) and the CRAC domain sequences, were aligned to show the conserved motif -L/V-(X)(1-5)-Y-(X)(1-5)-R/K-, which is highlighted in red letters with black background (except tryptophan, W). Residues that are in 80% or more conserved or 60% or more conserved are highlighted in green letters with grey background or in black letters with grey background, respectively. The categories of the sequence origins are indicated on the right, and the name of each sequence used in the alignment is indicated on the left.
(TIF)

Figure S2 **Sequence alignment of human and mouse Cyp11A1s and Cyp11B1s with the unique mitochondrial CYP enzyme, CYP44A1, in *C. elegans*.** Arrows indicate the putative cleavage site for CYP11A1 (red) and CYP44A1 (green). The CRAC domain (-L/V-(X)(1-5)-Y-(X)(1-5)-R/K-) and the F-G loop are highlighted. The amino acid residues important for the cholesterol access channel, recognition site for substrate entry, and substrate binding are indicated with red triangles as shown previously (Lewis et al., 1998, J Steroid Biochem Mol Biol 66: 217-233) and in the brown dots as shown in a recent report (Strushkevich et al., 2011, Proc Natl Acad Sci USA 108: 10139-10143). The mitochondrial CYP enzymes conserved motif C-GRR--E is also indicated.
(TIF)

Figure S3 **Phylogenetic analysis of MAPK3 (ERK1/2) using NJ tree.** The phylogenetic tree for animal MAPK3-related sequences. Gene duplication events in vertebrates are indicated by arrows. The circle phylogenetic tree on the right includes the top 500 related sequences retrieved from GenBank.
(TIF)

Figure S4 **Phylogenetic analysis of ACSL4 using NJ tree.** The phylogenetic tree for animal ACSL4-related sequences. The apparent gene duplication event in vertebrates is indicated by an arrow. The ACSL4-related sequences from plants and other family members are collapsed as indicated. The circle phylogenetic tree on the right includes the top 500 hit sequences retrieved from GenBank.
(TIF)

Figure S5 **Phylogenetic analysis of ACOT2/3 sequences.** The top 500 sequences related to the mammalian ACOT2/3 were used in this analysis as shown in the circle phylogenetic tree (right). Animal ACOT2/3–related sequences were subtracted for further analysis in the NJ tree (left), illustrating the evolutionary relationships of the ACOTS2/3 to ACOT4, ACOT6, BAAT and AAT2. Gene duplication events are indicated by arrows. Bootstrap values greater than 50% are indicated. ACOT4 or 6, acyl-CoA thioesterase 4 or 6; BAAT, bile acid CoA: amino acid N-acyltransferase (glycine N-choloyltransferase); AAT2, Aortic aneurysm, familial thoracic 2.
(TIF)

Table S1 **Accession numbers of the genes used as queries in this study.**
(PDF)
