# Supplementary figures and images for: Evolutionary Origin of the Mitochondrial Cholesterol Transport Machinery Reveals a Universal Mechanism of Steroid Hormone Biosynthesis in Animals
Source: PLoS One. 2013 Oct 4;8(10):e76701. doi: 10.1371/journal.pone.0076701 (PMC3790746; doi:10.1371/journal.pone.0076701)

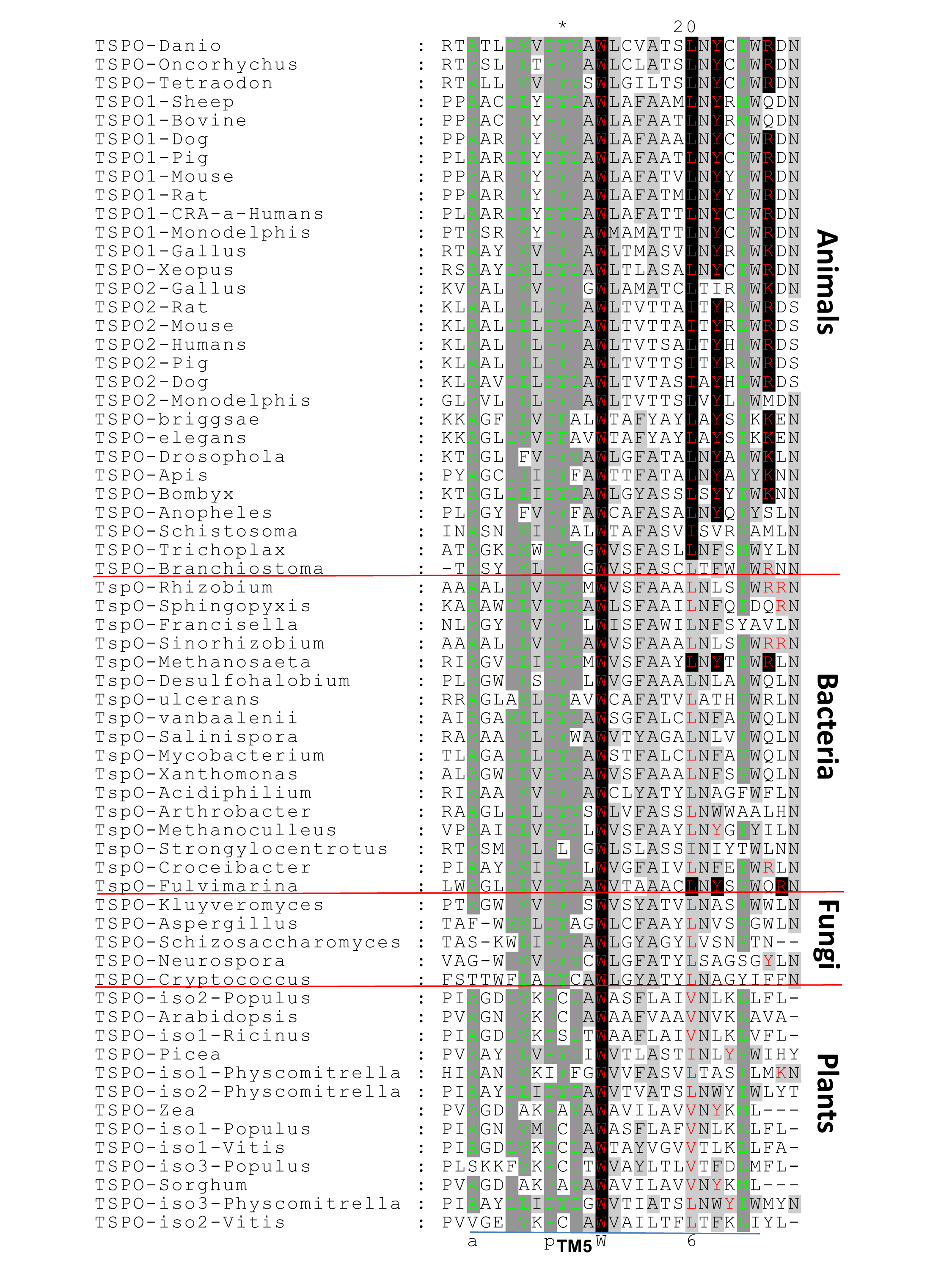

Supplement: Figure S1 — Sequence alignment of TSPO CRAC domains. Partial sequences, including transmembrane 5 (TM5) and the CRAC domain sequences, were aligned to show the conserved motif -L/V-(X)(1-5)-Y-(X)(1-5)-R/K-, which is highlighted in red letters with black background (except tryptophan, W). Residues that are in 80% or more conserved or 60% or more conserved are highlighted in green letters with grey background or in black letters with grey background, respectively. The categories of the sequence origins are indicated on the right, and the name of each sequence used in the alignment is indicated on the left. (TIF) [file pone.0076701.s001.tif]

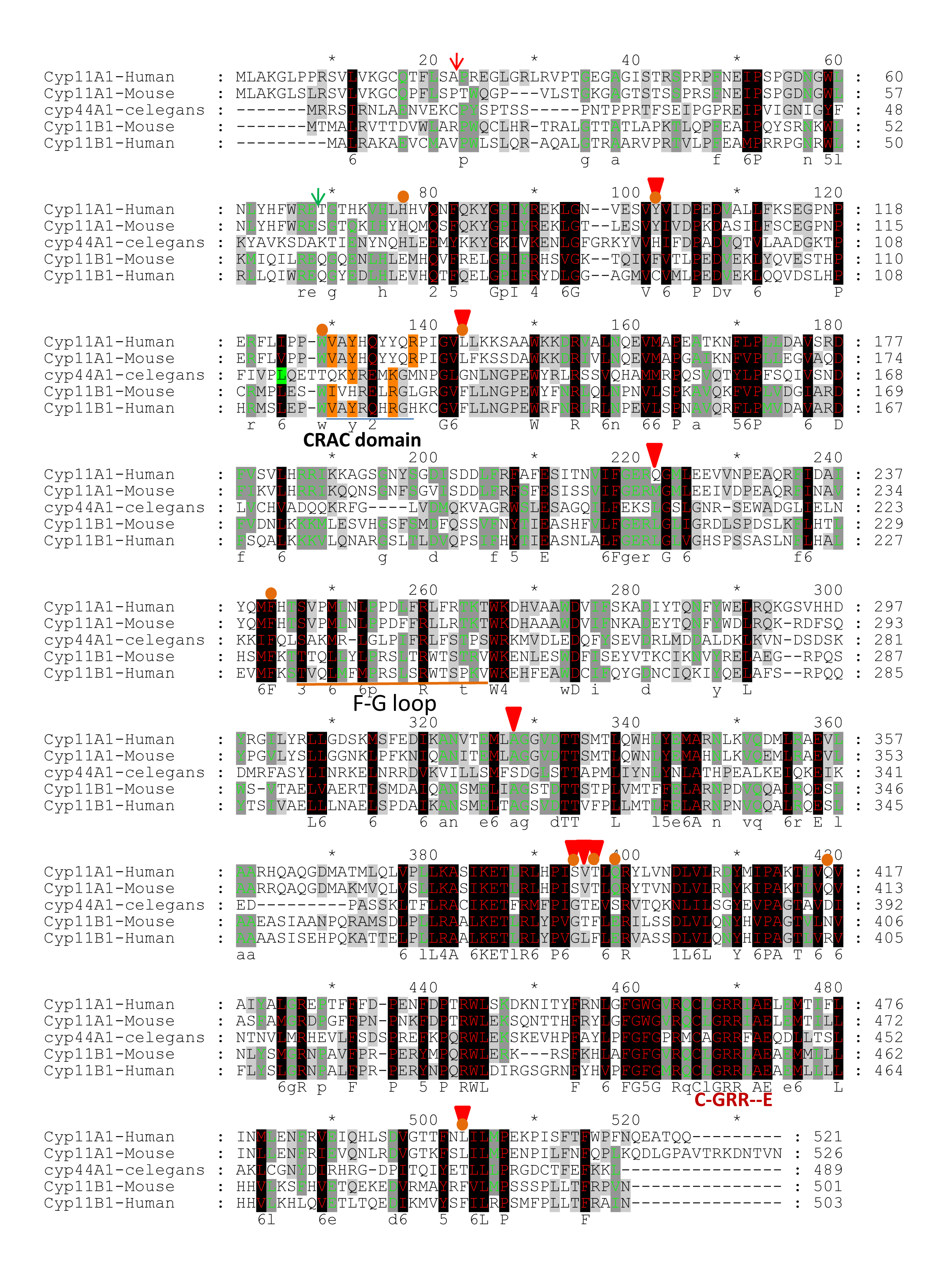

Supplement: Figure S2 — Sequence alignment of human and mouse Cyp11A1s and Cyp11B1s with the unique mitochondrial CYP enzyme, CYP44A1, in C. elegans. Arrows indicate the putative cleavage site for CYP11A1 (red) and CYP44A1 (green). The CRAC domain (-L/V-(X)(1-5)-Y-(X)(1-5)-R/K-) and the F-G loop are highlighted. The amino acid residues important for the cholesterol access channel, recognition site for substrate entry, and substrate binding are indicated with red triangles as shown previously (Lewis et al., 1998, J Steroid Biochem Mol Biol 66: 217-233) and in the brown dots as shown in a recent report (Strushkevich et al., 2011, Proc Natl Acad Sci USA 108: 10139-10143). The mitochondrial CYP enzymes conserved motif C-GRR--E is also indicated. (TIF) [file pone.0076701.s002.tif]

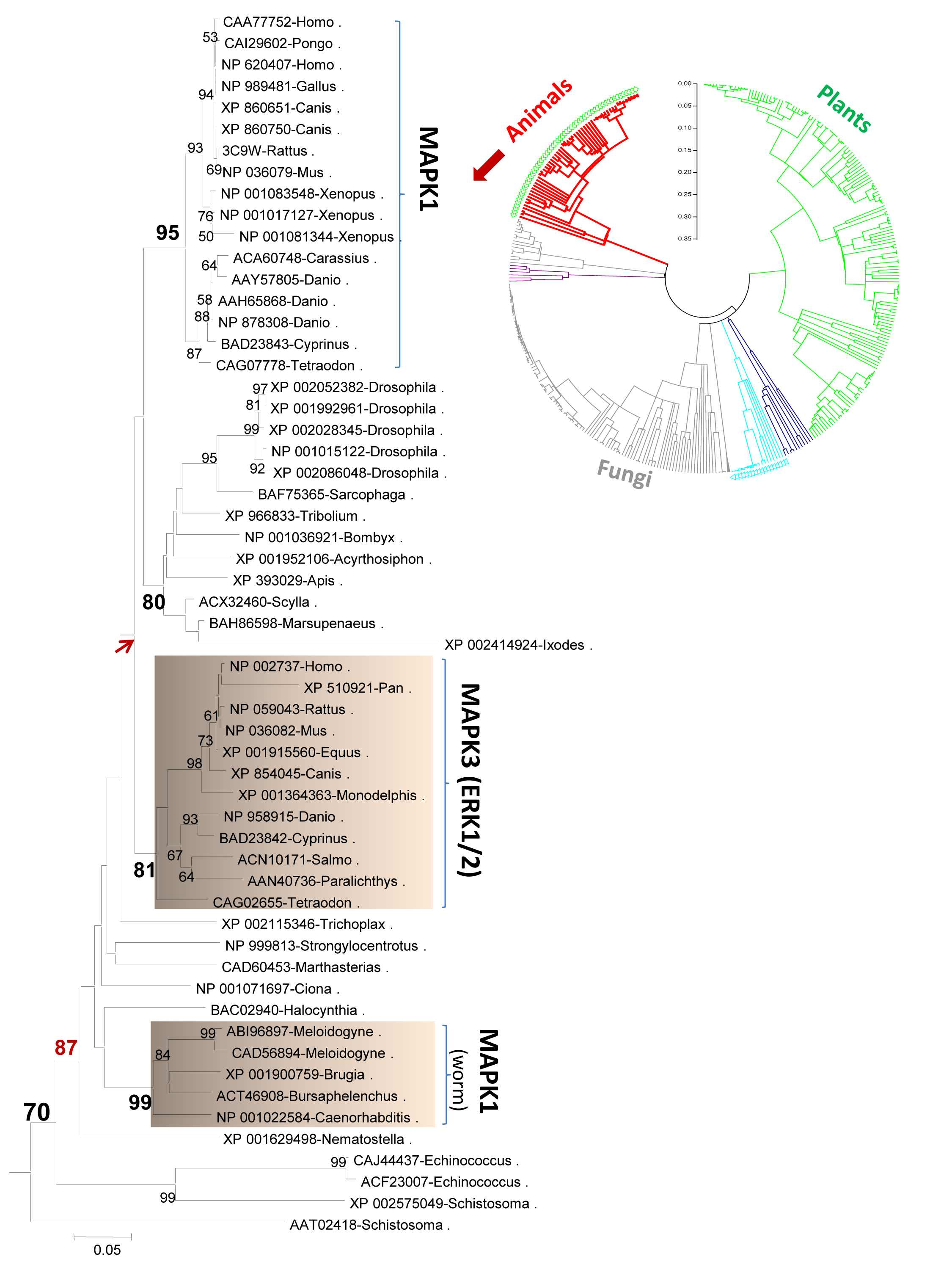

Supplement: Figure S3 — Phylogenetic analysis of MAPK3 (ERK1/2) using NJ tree. The phylogenetic tree for animal MAPK3-related sequences. Gene duplication events in vertebrates are indicated by arrows. The circle phylogenetic tree on the right includes the top 500 related sequences retrieved from GenBank. (TIF) [file pone.0076701.s003.tif]

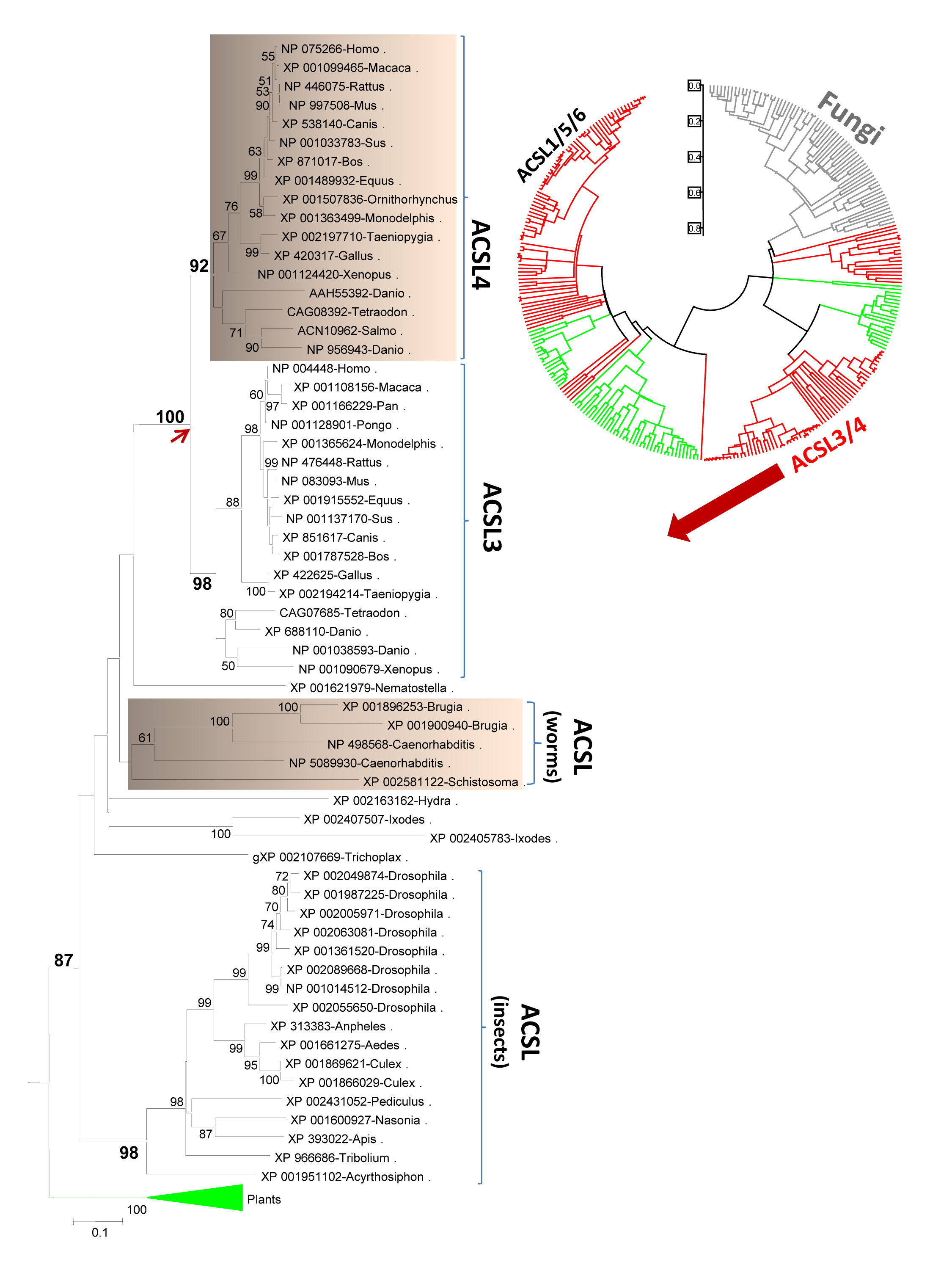

Supplement: Figure S4 — Phylogenetic analysis of ACSL4 using NJ tree. The phylogenetic tree for animal ACSL4-related sequences. The apparent gene duplication event in vertebrates is indicated by an arrow. The ACSL4-related sequences from plants and other family members are collapsed as indicated. The circle phylogenetic tree on the right includes the top 500 hit sequences retrieved from GenBank. (TIF) [file pone.0076701.s004.tif]

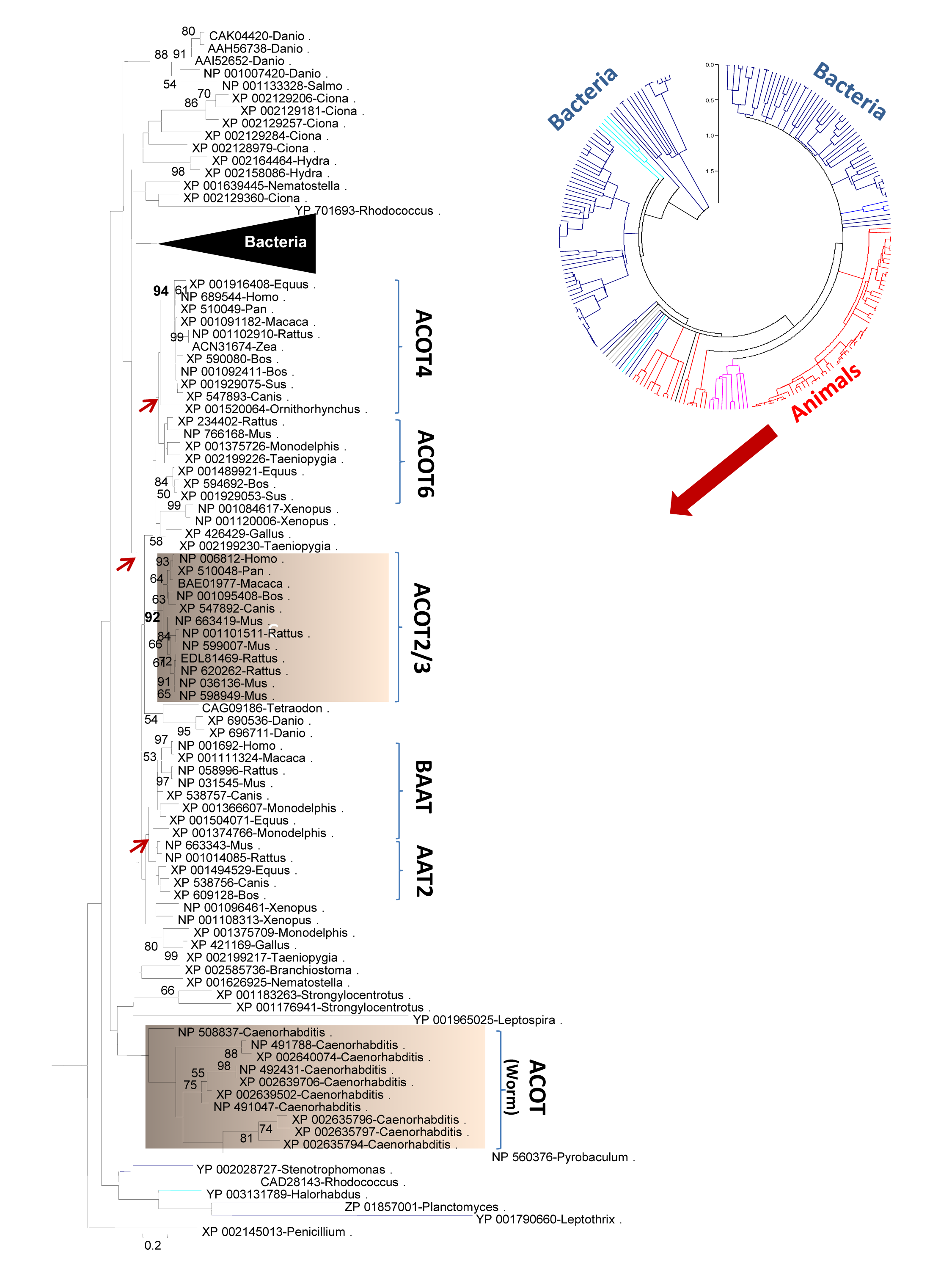

Supplement: Figure S5 — Phylogenetic analysis of ACOT2/3 sequences. The top 500 sequences related to the mammalian ACOT2/3 were used in this analysis as shown in the circle phylogenetic tree (right). Animal ACOT2/3–related sequences were subtracted for further analysis in the NJ tree (left), illustrating the evolutionary relationships of the ACOTS2/3 to ACOT4, ACOT6, BAAT and AAT2. Gene duplication events are indicated by arrows. Bootstrap values greater than 50% are indicated. ACOT4 or 6, acyl-CoA thioesterase 4 or 6; BAAT, bile acid CoA: amino acid N-acyltransferase (glycine N-choloyltransferase); AAT2, Aortic aneurysm, familial thoracic 2. (TIF) [file pone.0076701.s005.tif]
