# Supplementary material for: Evolutionary Origin of the Mitochondrial Cholesterol Transport Machinery Reveals a Universal Mechanism of Steroid Hormone Biosynthesis in Animals
Source: PLoS One. 2013 Oct 4;8(10):e76701. doi: 10.1371/journal.pone.0076701 (PMC3790746; doi:10.1371/journal.pone.0076701)
Supplement: Table S1 — Accession numbers of the genes used as queries in this study. (PDF) [file pone.0076701.s006.pdf]

Table S1.

Table S1. Accession numbers of the genes used as queries in this study

Abbreviation[a]	Species	Genbank Accession No.
TSPO	*Homo sapiens*	NP_000705
	Mus musculus	NP_033905
STAR/STARD1	*Homo sapiens*	NP_000340
	Mus musculus	NP_035615
ACBD3/PAP7	*Homo sapiens*	NP_073572
	Mus musculus	NP_573488
CYP11A1	*Homo sapiens*	NP_000772
	Mus musculus	NP_062753
ACBD1/DBI	*Homo sapiens*	NP_001033088
	Mus musculus	NP_065438
PRKAR1A	*Homo sapiens*	NP_002725
	Mus musculus	NP_068680
ATAD3	*Homo sapiens*	NP_001164006
	Mus musculus	NP_849534
MAPK3/ERK1/2	*Homo sapiens*	NP_002740
	Mus musculus	NP_036082
ACSL4	*Homo sapiens*	NP_075266
	Mus musculus	NP_997508
ACOT2/3	*Homo sapiens*	NP_006812
	Mus musculus	NP_598949
VDAC1	*Homo sapiens*	2K4T
	Mus musculus	3EMN
ANT1	*Homo sapiens*	NP_001142
	Mus musculus	NP_031476

a, TSPO, translocator protein (18kDa), or benzodiazapine receptor (peripheral; BZRP or PBR); STAR/STARD1, steroidogenic acute regulatory protein or START domain containing 1; ACBD3/PAP7, acyl-CoA binding domain containing 3 or PBR- and PKA-associated protein 7; CYP11A1, cytochrome P450, family 11, subfamily A, polypeptide 1; ACBD1/DBI, acyl-CoA binding domain containing 1 or diazepam binding inhibitor (GABA receptor modulator, acyl-CoA binding protein); PRKAR1A, protein kinase, cAMP-dependent, regulatory, type I, alpha; ATAD3, ATPase family, AAA domain containing 3A; MAPK3/ERK1/2, mitogen-activated protein kinase 3 or extracellular signal-regulated kinase ½; ACSL4, acyl-CoA synthetase long-chain family member 4; ACOT2/3, acyl-CoA thioesterase 2/3; VDAC1, voltage-dependent anion channel 1; ANT1, adenine nucleotide translocator 1 or solute carrier family 25 (mitochondrial carrier; adenine nucleotide translocator), member 4.
